# A Comparative Structural Bioinformatics Analysis of the Insulin Receptor Family Ectodomain Based on Phylogenetic Information

**DOI:** 10.1371/journal.pone.0003667

**Published:** 2008-11-07

**Authors:** Miguel E. Rentería, Neha S. Gandhi, Pablo Vinuesa, Erik Helmerhorst, Ricardo L. Mancera

**Affiliations:** 1 Western Australian Biomedical Research Institute and School of Biomedical Sciences, Curtin University of Technology, Perth, Western Austrailia, Australia; 2 School of Pharmacy, Curtin University of Technology, Perth, Western Austrailia, Australia; 3 Centro de Ciencias Genómicas, Universidad Nacional Autónoma de México, Cuernavaca, Morelos, Mexico; Center for Genomic Regulation, Spain

## Abstract

The insulin receptor (IR), the insulin-like growth factor 1 receptor (IGF1R) and the insulin receptor-related receptor (IRR) are covalently-linked homodimers made up of several structural domains. The molecular mechanism of ligand binding to the ectodomain of these receptors and the resulting activation of their tyrosine kinase domain is still not well understood. We have carried out an amino acid residue conservation analysis in order to reconstruct the phylogeny of the IR Family. We have confirmed the location of ligand binding site 1 of the IGF1R and IR. Importantly, we have also predicted the likely location of the insulin binding site 2 on the surface of the fibronectin type III domains of the IR. An evolutionary conserved surface on the second leucine-rich domain that may interact with the ligand could not be detected. We suggest a possible mechanical trigger of the activation of the IR that involves a slight ‘twist’ rotation of the last two fibronectin type III domains in order to face the likely location of insulin. Finally, a strong selective pressure was found amongst the IRR orthologous sequences, suggesting that this orphan receptor has a yet unknown physiological role which may be conserved from amphibians to mammals.

## Introduction

Insulin and the insulin-like growth factors (IGFs) are homologous protein hormones that play distinct physiological roles in mammals and other animals. Whilst the former is the primary regulator of carbohydrate homeostasis and has effects on lipid and protein metabolism [1], [2] the latter stimulate cell growth, replication and differentiation [3], [4]. The mechanism of action of these hormones is mediated by their specific binding to the Insulin Receptor (IR) or the type 1 Insulin-like Growth Factor Receptor (IGF1R) [5].

The IR and IGF1R, along with the IR-Related Receptor (IRR) [6], [7], [8] form subclass II of the Receptor Tyrosine Kinase (RTK) superfamily [9], and unlike the other members which dimerise or oligomerise upon ligand binding, the IR family members are pre-formed covalently-linked homodimers (α_2_β_2_) consisting of several structural domains [10]. It is possible that these receptors also function as heterodimers, since IR/IGF1R hybrids have been found in all tissues expressing both receptors [11], [12] but their physiological role remains unknown.

The IR is expressed in two isoforms IR-A (exon 11−) and IR-B (exon 11+) [13] that display differential kinase activity [14]. Both isoforms have similar affinity for insulin [15]. However, IR-A shows considerably higher affinity for IGF-1 and particularly for IGF-2 than IR-B [16], and has been implicated together with the IGF1R in malignant transformation [17], [18].

Although no ligand has yet been associated to the IRR, its expression in a variety of tissues including kidney, heart, liver and pancreas has been reported [19]. Likewise, single and combined IR family knockout models in mice were recently established [20], suggesting that the IRR could function as an auxiliary member of the IR family, a role that may extend to other co-expressed recognition molecules, such as the *TrkA* receptor [21], [22].

IR family members are synthesised as single-chain pre-proreceptors, which are then glycosylated, folded, dimerised and processed to produce the mature α_2_β_2_ receptors [23]. Each receptor consists of an ectodomain, a transmembrane segment and an intracellular tyrosine kinase. The ectodomain comprises two leucine-rich repeat structural domains (usually referred to as L1 and L2) separated by a cysteine-rich (CR) region [24], followed by three fibronectin type III domains (FnIII-1, FnIII-2 and FnIII-3) [25], [26], the second of which features an insert domain (ID) that contains the site of cleavage between the α and β subunits and the alternatively spliced exon 11.

The structural determination of the first three domains of the IGF1R was reported in 1998 [27], facilitating the subsequent mapping of functional regions to the L1 and CR domains that contribute to ligand binding and affinity through alanine scanning mutagenesis [28], [29], [30], [31], chimeric receptor constructs [32], [33], [34] and cross-linking [35], [36], [37] studies involving both IR and IGF1R.

Attempts to obtain insights into the ectodomain arrangement and ligand binding of the IR have included a three-dimensional reconstruction based on images obtained by electron cryomicroscopy [38]. The three-dimensional structure of the intact IR dimer ectodomain was recently determined by X-ray crystallography, revealing an “inverted V” arrangement, wherein the first three domains (L1-CR-L2) form one leg and the three FnIII domains make up the other leg in each monomer [39]–[40]. The two monomers are located in an anti-parallel orientation and are linked by a disulphide bond at Cys 524. Similarly, there is a second inter-α-chain disulphide bond at the Cys 682, Cys 683 and Cys 685 triplet in the insert domain, and the α and β chains are linked within the monomer by a single disulphide bond at Cys647–Cys860. The dimer crystal structure features two potential ligand binding sites and helps rationalise many characteristics of ligand-receptor binding, such as the existence of both low- (site 1) and high-affinity (site 2) binding sites and negative cooperativity, as inferred from Scatchard plots [41], [42]. The domain arrangement in the ectodomain crystal structure also suggests that receptor binding site 2 involves one or more of the FnIII domains, as opposed to a previously proposed model that suggested that the first three domains of each monomer jointly participate in insulin binding [38].

A crystal structure of only the first three domains of the IR was also recently obtained, and a possible model of insulin binding to the L1 domain was proposed [43]. There is evidence that the B chain C-terminal of insulin contacts the insert domain of the IR [37], [44], [45], presumably upon a conformational change of insulin [37], [46], [47], [48]. However, the insert domain could not be crystallised, presumably due to its disordered conformation [39]. Hence, the proposed model of the binding of insulin omitted the C-terminal portion of its B chain. In the absence of a crystal structure of the complex between insulin and its receptor, further investigation is needed to determine the contribution of L2 and the three FnIII domains to insulin binding and receptor ligand specificity.

Recently, studies involving the construction of chimeric receptors have shown that there is a significant contribution of L2 and particularly of FnIII-1 to insulin binding [49], but it was not possible to determine the specific residues on these domains that may be involved in contacting insulin. In order to map those and other possible regions in the IR contributing to insulin binding, we have performed a comparative structural bioinformatics analysis of the insulin receptor family ectodomain based on phylogenetic information.

Biological evolution has recorded vast and highly precise information in genetic sequences. For this reason, amino acid sequences are a powerful source of information for predicting functional regions of proteins by analysing conservation patterns. It is known that regions directly involved in biochemical functions, such as binding surfaces, experience different selection pressure from other regions on the surface of proteins [50]. In the same way, non-polar amino acids in the interior of a protein may be conserved due to structural and stability constraints as hydrophobic interactions are considered to be the driving force of protein folding [51], [52]. Although mutation rates and conservation scores can be estimated for each amino acid position of a protein sequence from a multiple sequence alignment, it is necessary to correlate these data with their corresponding location in the three-dimensional structure, since residues that are distant in sequence, can be found in close proximity in the folded protein.

In view of the evidence that associates the FnIII domains of the IR to insulin binding, we have attempted to map the evolutionarily conserved regions of these domains in order to predict those specific residues that might contact insulin. Homologous amino acid sequences of the IR family ectodomain in mammals, birds, amphibians and fish were retrieved from public databases through a BLAST search, and were then classified into three different orthologous sets corresponding to the IR, IGF1R and IRR. Each set was subsequently aligned and evolutionary conservation scores at each amino acid position were calculated for the IR and IGF1R using the Rate4Site algorithm [53]. The resulting scores were categorised into different conservation grades and projected onto the three-dimensional X-ray structures of the IR family, as available from the Protein Data Bank (PDB) [54]. We have aimed to obtain a precise and detailed model of the ligand-binding interactions and to identify the residues that are responsible for ligand recognition specificity amongst paralogous receptors. This knowledge may be used in future for the rational design of drugs to treat diseases such as diabetes and cancer.

Through this amino acid conservation analysis we reconstructed the phylogeny of the IR family and predicted with significant accuracy the location of the well studied binding site 1 of the IGF1R and IR. We have also predicted the potential location of insulin binding site 2 on the FnIII-1 and FnIII-2 surface of the IR. At the same time, we could not identify a conserved surface on the L2 domain that may contact the ligand. We have also suggested a possible mechanical trigger of the activation of the IR on the basis of normal modes analysis of the low-frequency vibrations of this receptor. Finally, a strong selective pressure was found amongst the IRR orthologous sequences, suggesting that this orphan receptor has a yet unknown physiological role which may be conserved from amphibians to mammals.

## Results and Discussion

### Phylogenetic Analysis of the IR family

Invertebrates possess only a single homologous receptor of the IR family [55]. In addition to its function in the regulation of metabolism, insulin signalling in *Drosophila Melanogaster* (fruit fly) and *Caenorhabditis elegans* (roundworm) has a role in lifespan and reproduction control [56], [57], whilst in *Apis Mellifera* (honeybee) it is involved in caste determination and differentiation [58].

A significant step in the evolution of the IR family has been the transition from a single invertebrate IR that regulates both growth and metabolism to two different and specialised receptors that are able to recognise and discriminate their specific ligands: the IR and the IGF1R in vertebrates [57], [59]. Studies in primitive vertebrates suggest that gene duplication would have occurred early in vertebrate evolution [60], [61].

**Figure 1:**
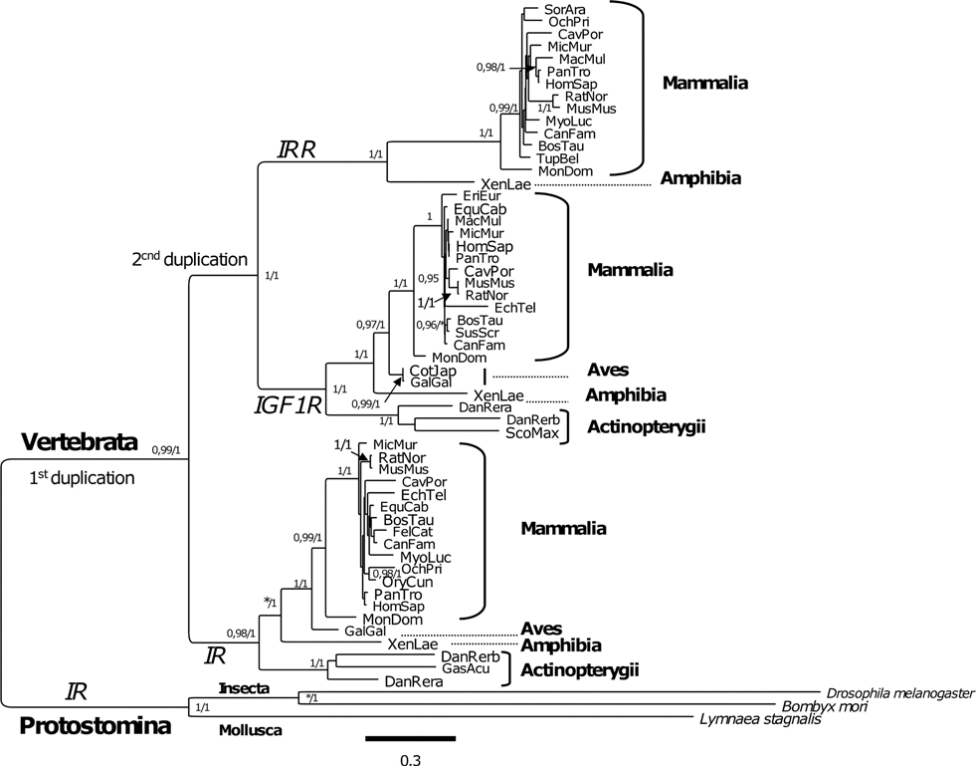
Evolution of the IR family. Maximum likelihood phylogeny of the IR family ectodomain inferred from amino acid sequences. The tree shown was the best one found (ln*L* = −30836.65814) amongst 41 independent tree searches (see Materials and Methods) for 55 vertebrate and 3 invertebrate IR family ectodomain homologues. The numbers on the bipartitions indicate the ML SH-like *P* values/Bayesian posterior probability support values. Only significant (*P*≥0.95) values are shown. An asterisk (*) indicates that the bipartition was not significantly supported (*P*<0.95) either by the highly conservative SH-like branch significance test [103] used to compute bipartition robustness under the ML criterion, or by the more liberal Bayesian posterior probabilities. NCBI taxonomic ranks are provided for some clades. The scale indicates the number of expected substitutions per site under the best fitting JTT+G model (shape parameter α = 0.865), which had a posterior probability of 1.

In order to study the phylogenetic relationships between the distinct members of the IR family, a multiple sequence alignment (MSA) including the 55 vertebrate ectodomain sequences listed in Table 1, plus three IR family invertebrate homologous ectodomain sequences, was constructed using MUSCLE [62]. The first 53 residues of the *Bos taurus* 1GF1R protein (XP_606794.3) were excluded because they were not homologous to the N-termini of the other mammalian orthologues, as seen in the MSA and confirmed by BLASTP searches. Bayesian and maximum likelihood tree searches under the best-fitting model were performed as described in the Methods section. Figure 1 shows the best ML tree found under the substitution model with highest posterior probability (JTT+G). Great model selection confidence was also found for each of three individual sets (IR, IGF1R and IRR) of orthologous sequences when evaluated with ProtTest [63]. The model selected was in all cases JTT+G, with an Akaike weight ranging between 0.74 and 0.75 (the analysis is provided in Supplementary File S1). All the deeper bipartitions of the phylogeny were significantly supported (≥0.95 SH-like *P* values or Bayesian posterior probabilities) by both types of tree searches, as indicated on Figure 1. The two independent Bayesian MC^3^ runs yielded identical topologies (Robinson-Fould distance = 0) which, when compared with that of the best-scoring ML tree (Figure 1), differed only in a few bipartitions. These corresponded to poorly resolved terminal clades which formed polytomies on the Bayesian tree (shown in Supplementary File S2). Therefore, both optimality criteria consistently and significantly support the hypothesis that the IRR has a closer relationship to the IGF1R as compared with the IR, suggesting that the IGF1R and IRR share a common ancestor and that they are the product of a second gene duplication event in the IR family evolution history (Figure 1). This is consistent with the fact that no orthologous IRR sequence could be found in fish genomes in this study. However, the possibility of a loss of the IRR paralogous in fish remains to be proved as it is not possible to establish with certainty that this occurred with current available data. Nevertheless, a posterior duplication of the IR and the IGF1R genes occurred in the zebra fish lineage, presumably as a product of whole-genome duplication, therefore giving origin to two different functional versions (a and b) of both the IR and the IGF1R [64]. Whilst both versions of the IGF1R are required for proper zebra fish embryonic growth, development and survival, IGF1Rb plays a considerably higher role in spontaneous muscle contractility and motoneuron development [65].

**Table 1:** Orthologous sequences and their accession numbers obtained from the GenBank and ENSEMBL databases.

Species	Common Name	IR	IGF1R	IRR
*Bos taurus*	Cow	XP_590552	XP_606794.3	XP_001254386
*Canis familiaris*	Dog	XP_542108.2	XP_545828.2	XP_547526
*Cavia porcellus*	Guinea Pig	ENSCPOG00000011692	ENSCPOP00000004859	AAA37044
*Coturnix japonica*	Japanese Quail		BAF73401	
*Danio rerio*	Zebra Fish	XP_690534.1, NP_001116701	NP_694500, NP_694501	
*Echinops telfairi*	Lesser Hedgehog Tenrec	ENSETEP00000014592	ENSETEP00000009987	
*Equus caballus*	Horse	XP_001496634	XP_001489815.1	
*Erinaceus europeus*	Western European Hedgehog		ENSEEUP00000012248	
Felis catus	Cat	ENSFCAP00000002790		
*Gallus gallus*	Chicken	XP_418250.2	NP_990363.1	
Gasterosteus aculeatus	Threespine Stickleback Fish	ENSGACP00000013853		
*Homo sapiens*	Human	AAA59174.1	AAB22215	NP_055030
*Macaca mulatta*	Rhesus Monkey		XP_001100407.1	XP_014528
*Microcebus murinus*	Mouse Lemur	ENSMICP00000010917	ENSMICP00000009223	ENSMICP00000008186
*Monodelphis domestica*	Gray Short-Tailed Opossum	XP_001377572.1	XP_001372725.1	ENSMODP00000020864
*Mus musculus*	Mouse	NP_034698.2	NP_034643.2	NP_035962
*Myotis lucifugus*	Bat	ENSMLUP00000012576		ENSMLUP00000009532
*Ochotonas princeps*	American Pika	ENSOPRP00000004065		ENSOPRP00000014561
*Oryctolagus cuniculus*	Rabbitt	AAR04440		
*Pan troglodytes*	Chimpanzee	XP_512324.2 & XP_512323.2	XP_001136377	XR_025504
*Rattus norvegicus*	Rat	EDL74923	NP_434694.1	XP_001068054
*Sorex araneus*	Common Shrew			ENSSARP00000011012
*Scophthalmus maximus*	Turbot fish		CAA12278	
*Sus scrofa*	Pig		NP_999337.1	
*Tupaia belangeri*	Northern Treeshrew			ENSTBEP00000014243
*Xenopus laevis*	African Clawed Frog	NP_001081702	NP_001081734.1	MP_001083465

Both the IR and IRR contain an additional exon (exon 11) with respect to IGF1R. In the IR, it gives rise to the two different IR-A and IR-B isoforms, whereas in the IRR this exon is constitutively expressed as part of the receptor. A recent study traced the presence of the alternatively spliced exon 11 of the IR, showing that it is a novel acquisition of mammals [66]. Furthermore, given the highly divergent sequences and the phylogenetic relationship of this exon amongst the IR family members, it was also proposed that both exons were independently acquired by each paralogous gene [66].

A possible selective advantage conferred by the evolutionary acquisition of exon 11 by the IR is explained by the fact that isoform B is predominantly expressed in insulin target tissues that are involved in glucose homeostasis [13], [67], which may be the consequence of a more specialised function as a metabolic receptor.

### IR and IGF1R Conservation

With the increasing amounts of DNA and amino acid sequences available in public databases, performing comparative multi-species phylogenomics studies is now feasible. In the case of families of genes, it is possible to study the evolution and divergence of paralogous and orthologous proteins. This information, along with protein structures, when available, can be used to computationally predict functional regions of proteins.

In this study, the MSAs corresponding to the IR and IGF1R ectodomain orthologous sets were used to estimate conservation scores with the Rate4Site algorithm under the maximum-likelihood model. The conservation scores were projected onto the crystal structures of the IR family, as available from the Protein Data Bank (PDB IDs: 2DTG, 1IGR, 2HR7), by using the ConSurf server [68]. The conservation score at a particular position corresponds to its evolutionary rate. Whilst some positions evolve rapidly and are commonly referred to as “variable”, other positions evolve slowly and are referred to as “conserved”. The variations in the rate of conservation correspond to different levels of purifying selection acting on each site. Purifying selection is expected to be higher at structurally and functionally critical positions, such as protein-protein binding surfaces.

**Figure 2:**
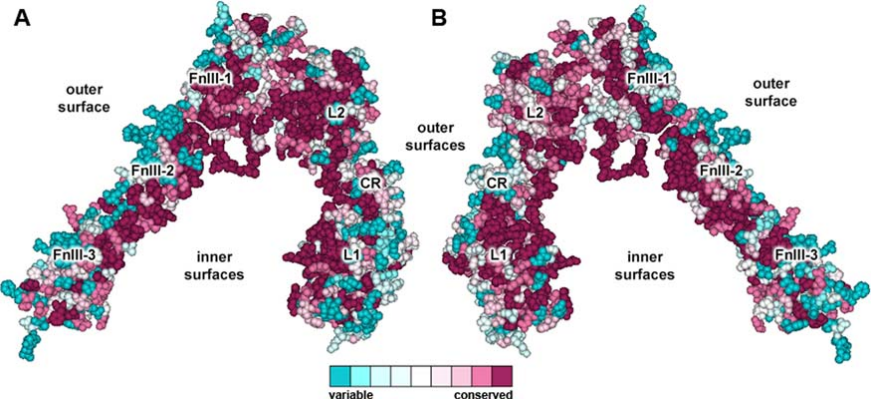
Amino acid conservation in the IR ectodomain monomer. Amino acid conservation scores were classified into nine levels. This figure shows the general conservation of the two faces of a single IR ectodomain monomer: facing towards both (A) outside and (B) inside of the dimer. The colour scale for residue conservation is indicated in the figure. The molecular coordinates were taken from PDB structure 2DTG.

In the IR dimer structure, the first three domains of each monomer are packed against the three FnIII domains of the other monomer, in such a way that the L1 and L2 domains from each monomer correspondingly interact with the FnIII-3 and the FnIII-1 domains of the other monomer. Figure 2 shows the degree of residue-specific conservation in the structure of an IR monomer ectodomain. The “inner” surface of the monomer exhibits a considerable higher conservation than the “outer” surface. This is, to a certain extent, due to the interactions between the L2 and FnIII-1 domains from the same monomer, which might be an indispensable requirement for the monomer to adopt the inverted “V” conformation. Furthermore, the conserved surfaces also correspond to the regions involved in monomer-monomer interactions.

**Figure 3:**
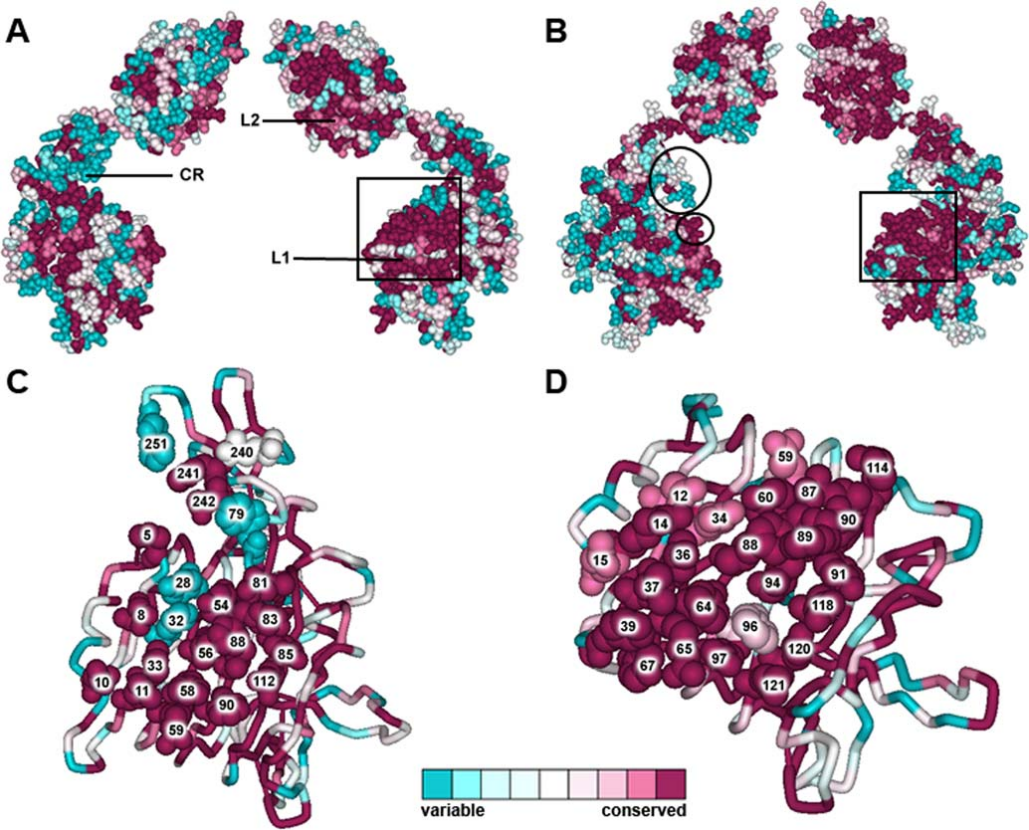
Comparison of the overall amino acid conservation of the first three domains of the IR and IGF1R and their proposed ligand binding sites. Amino acid conservation in the L1-CR-L2 domains of the (A) IGF1R and (B) IR. The rectangles indicate the regions that are believed to be involved in ligand binding and the circled regions in B indicate the location of the major specificity regions between the IR and IGF1R.[43] (C) A proposed binding site on the IGF1R surface is shown (available supporting mutagenesis data is listed in Table 3). (D) Insulin binding surface on the L1 domain (mutagenesis data is listed in Table 2). Variations at non-strictly conserved positions on the binding surfaces are indicated. Surfaces in C and D are in agreement with previous ligand binding models.[43], [78]. The figures are based on the coordinates of the IGF1R (PDB code 1IGR) and IR (PDB code 2HR7).

A comparison of the overall conservation of the first three domains of both the IGF1R and IR is shown in Figures 3A and B. The “inner” surface of the L1 domain of the IR features a conserved surface (shown in Figure 3D) formed by residues Asp12, Arg14, Asn15, Gln34, Leu36, Leu37, Phe39, Tyr60, Leu62, Phe64, Arg65, Tyr67, Leu87, Phe88, Phe89, Asn90, Tyr91, Val94, Phe96, Glu97, Arg114, Arg118, Glu120 and Lys121, which, on the basis of mutagenesis data, is likely to be involved in ligand binding. Mutagenesis data was extracted from the Receptors for Insulin and Insulin-like Molecules (RILM) online database [69] and is listed in Table 2. The only residues that are not strictly conserved are Asp12, Asn15, Asp59 and Phe96. Likewise, the IGF1R features a similar conserved region comprising residues Pro5, Asp8, Asn11, Tyr28, His30, Leu32, Leu33, Tyr54, Leu56, Phe58, Arg59, Trp79, Leu81, Phe82, Tyr83, Tyr85, Val85, Val88, Asn90, Arg112, Arg240, Phe241, Glu242 and Phe251, that would serve as the IGF-1 binding site. Figure 3C displays their conservation rates and the mutagenesis data is listed in Table 3.

**Table 2:** Alanine scanning and other mutagenesis data available for conserved residues on the L1 binding interface of IR (according to the RILM database).

IR Residue	Mutation	Reduction in affinity for insulin	Reference
Asp12	D12A	5 to 6.2-fold	[30], [109]
Arg14	R14A	+500-fold	[109]
Asn15	N15A	134-fold	[109]
Gln34	Q34A	3.2-fold	[109]
Leu36	L36A	1.6-fold	[109]
Leu37	L37A	20-fold (IR-A), 40-fold (IR-B)	[31]
Phe39	F39A	10-fold	[109]
Asp59	D59G^1^	4-fold	[110]
Tyr60	Y60A	Not secreted^2^	[30]
Leu62	L62P^1^	Insulin-resistant diabetes mellitus	[110]
Phe64	F64A	+500-fold	[109]
Arg65	R65A	No effect	[30]
Tyr67	Y67A	3.2-fold	[109]
Leu87	L87A	85% reduction	[111]
Phe88	F88A	No significant effect	[112]
Phe89	F89A	4.8-fold	[109]
Tyr91	Y91A	3.6-fold	[109]
Val94	V94A	3 to 10-fold	[31]
Phe96	F96A	Not secreted^2^	[30]
Glu97	E97A	3 to 10-fold	[31]
Arg114	-----	Data not available	
Arg118	-----	Data not available	
Glu120	E120A	3 to 10-fold	[31]
Lys121	K121A	3 to 10-fold	[31]
Thr704	T704A	More than 500-fold	[109]
Phe705	F705A	More than 500-fold	[109]
Glu706	E706A	More than 500-fold	[109]
Asp707	D707A	7 to 16-fold in the A isoform	[109]
Tyr708	Y708A	218-fold	[109]
Leu709	L709A	70-fold	[109]
His710	H710A	More than 500-fold	[109]
Asn711	N711A	52-fold	[109]
Val713	V713A	7 to 16 fold in the A isoform	[109]
Phe714	F714A	182-fold	[109]
Val715	V715A	8-fold	[109]

1 Naturally occurring mutations.

2 When expressed transiently in adenovirus-transformed human embryonic kidney cells.

**Table 3:** Alanine scanning and other mutagenesis data available for conserved residues on the L1 and the CR binding interfaces of the IGF1R (according to the RILM database).

IGF1R residue	Mutation	Reduction in affinity for IGF-1	Reference
Pro5	-----	Data not available	
Asp8	D8A	9-fold	[29]
Asn11	N11A	7.5-fold	[29]
Tyr28	Y28A	4.5-fold	[29]
His30	H30A	4.5-fold	[29]
Leu32	L32A	No significant effect	[113]
Leu33	L33A	6-fold	[29]
Tyr54	-----	Data not available	
Leu56	L56A	5-fold	[29]
Phe58	F58A	3-fold	[29]
Arg59	R59A	5-fold	[29]
Trp79	W79A	3-fold	[29]
Leu81	L81A	No significant effect	[29]
Phe82	F82A	No significant effect	[29]
Tyr83	Y83A	No significant effect	[29]
Tyr85	Y85A	No significant effect	[29]
Val88	V88A	No significant effect	[29]
Asn90	N90A	No significant effect	[29]
Arg112	R112A	No significant effect	[29]
Arg240	R240A	2-fold	[29]
Phe241	F241A	6-fold	[29]
Glu242	E242A	4-fold	[29]
Phe251	F251A	2-fold	[29]
Phe692	F692A	30-fold	[29]
Glu693	E693A	10-fold	[29]
Asn694	N694A	10-fold	[29]
Leu696	L696A	20-fold	[29]
His697	H697A	10-fold	[29]
Asn698	N698A	10-fold	[29]
Ile700	I700A	20-fold	[29]
Phe701	F701A	Abolishes IGF1 binding	[29]

### Ligand-Receptor Binding

The physiologically active form of insulin is a monomer composed of two chains, an A chain of 21 amino acids and a B chain of 30 residues, linked by two disulphide bonds at A7–B7 and A20–B19. An additional intra-chain disulphide bridge is situated between residues A6 and A11 [70], [71]. IGF-1 and IGF-2 are homologous peptides structurally related to insulin. The most important structural difference of the IGFs with respect to insulin is that they are single chain polypeptides that contain four structural domains: A, B, C and D [4], [72].

The most widely accepted model of insulin binding suggests that the insulin molecule comprises two separate binding surfaces, denominated as site 1 and site 2 [73]. These surfaces cross-link two different binding sites on the ectodomain of the IR. The classical binding surface of insulin (site 1) overlaps with the hexamer-forming surface and involves residues A1–A3, A5, A19, A21 as well as B12, B16, B23, B24 and B25 [73], [74], whereas site 2 overlaps with the dimer-forming surface and comprises residues A12, A13, A17, B10, B13 and B17 [42]. Moreover, it has been demonstrated that high-affinity IGF1 binding to the IGF1R involves the interaction between the IGF1 C-domain and the Cys-rich region of the IGF1R [75]. The lack of the C-domain greatly explains the low affinity of insulin for the IGF1R [75]. Nevertheless, an IGF1 analogue that binds with similar affinity to insulin for the IR was produced recently by introducing four insulin residues [76], which indicates that the IGF1 C-domain can be accommodated in the insulin binding site. This evidence is in agreement with previous experiments that showed that a single chain insulin analogue, wherein the A and B chains are connected by the C domain of IGF1, can bind to IR with the same affinity as wild-type insulin [77].

There is considerable evidence that a conformational change involving the C-terminal of insulin B chain occurs upon binding [37], [46], [47], [48]. The portion corresponding to residues B21–B30 is believed to move away from its contact with residues A1 and A2, in order to expose the hydrophobic “classic binding site” of insulin. Furthermore, it has been proposed that the N-terminal portion of the B chain (residues B1–B8) also experiences a change in conformation, from an extended and stable form, known as the T state to a less stable but more active form, known as the R state [47].

The conserved surfaces on the L1 and CR domains of the IGF1R and IR, when contrasted with the available mutagenesis data, reveal the strong correlation between the degree of evolutionary conservation of an amino acid position and its functional role, such as, in this case, its participation in a protein-protein binding interface. These binding site interfaces are in agreement with previous models of ligand binding and mutagenesis data [43], [78].

In order to identify those specific amino acid positions subjected to positive selection that might confer ligand-specificity to each paralogous receptor, we looked for divergent selection patterns at residues involved in ligand-binding. Interestingly, we found that residues Tyr28, His30, Trp79 and Arg240 of the IGF1R have diverged from their corresponding residues in the IR and IRR: His, Gln, Ser/Tre and His. This partly explains why these positions are less conserved than the rest of residues that contribute to IGF-1 binding on the surface of the L1 domain, as can be appreciated from Figure 3C.

The insert domain (ID) has been shown to play a role in insulin binding. Cross-linking studies revealed that two consecutive insulin residues, PheB24 and PheB25, contact two different domains of the IR: L1 and ID, respectively [37]. Consequently, it is believed that the ID is in close juxtaposition to the L1 domain. Recently, complementation analysis showed that these interactions occurs as a result of a *trans* mechanism, in which the ID and the L1 domain that simultaneously contact insulin belong each to different monomers of the IR [79].

**Figure 4:**
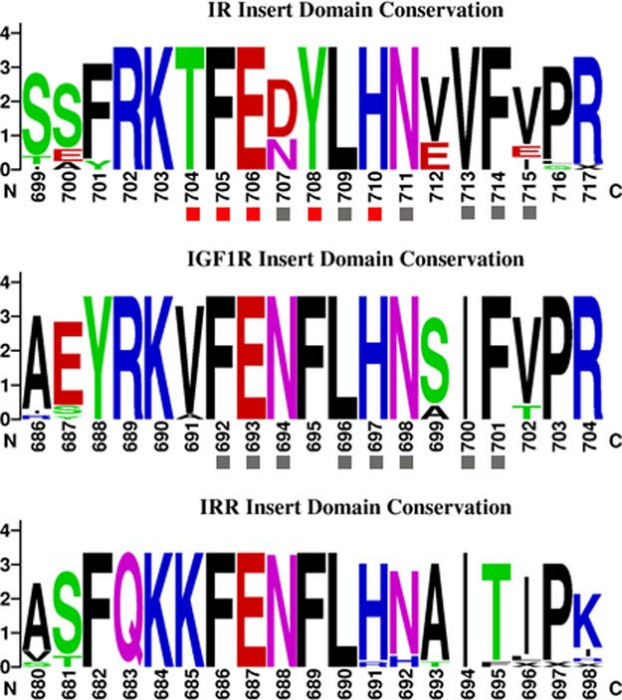
Conservation of the Insert Domain region involved in binding. Residues within the 700–715 fragment of the IR have been implicated in ligand binding. This logo representation also shows the corresponding residues in the IGF1R and IRR. Residues with a gray box result in considerable loss of binding when mutated to alanine, according to previous studies. Data is listed in Tables 2 and 3. Residues with a red box result in a 200- to 500-fold loss of binding.

Alanine scanning mutagenesis of the ID have indicated that residues Thr704, Phe705, Glu706, Tyr708, Leu709, His710, Asn711 and Phe714 display a considerable loss in insulin binding affinity upon mutation [31]. We have found that these residues show a strict conservation pattern in all 20 sequences of the IR used in this study, from fish to mammals, which correlates with their critical contribution to ligand binding. The corresponding region in the IGF1R is also involved in IGF1 binding. Individual mutation to alanine of residues Phe692, Glu693, Asn694, Leu696, His697, Asn698 and Ile700 resulted in a 10- to 30-fold loss in ligand binding, whilst mutation of Phe701 resulted in no detectable ligand binding. Figure 4 illustrates using a logo representation the evolutionary conservation of this region in the three orthologous receptors. It can be seen that most of the IR residues involved in binding are also evolutionary conserved in the IGF1R and IRR, suggesting that whilst this region contributes to ligand affinity, its contribution to ligand selectivity may be small.

A shortened IR, consisting of residues 1–601 and 650–719, displays the same insulin binding properties as the holoreceptor, suggesting that all residues needed for high affinity binding are located within these regions [80]. What is not clear yet, though, is whether this is also enough for triggering a conformational change of the receptor that ultimately leads to signal transduction.

**Figure 5:**
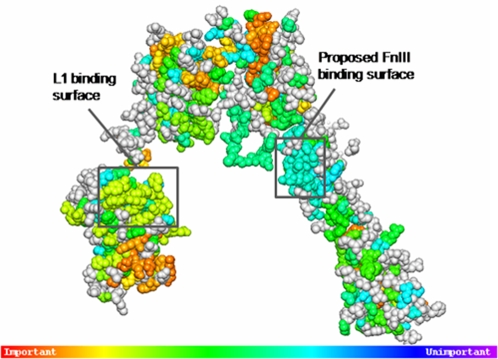
Functionally important residues in the IR predicted by Evolutionary Trace. Evolutionary Trace (ET) was performed on the 20 IR sequences in order to compare with ConSurf predictions. ET assigns a relative score of functional importance to each sequence residue. Residues predicted to be significantly important (rho≤2.8) are shown in the figure. Residues comprising both the known L1 ligand-binding surface and the proposed surface on FnIII display homogeneous functional scores, thus forming potential functional clusters. Likewise, residues implicated in structural stability were assigned high scores. The figure was generated with ET Viewer 2.0.

In this work, a region of conserved residues on the FnIII surface that face the proposed L1 domain binding site in the IR was identified. This region comprises residues Tyr507, Asn527, Trp529, Lys557, Pro558, Trp559, Ser596, Val597, Pro598, Leu599, Asp600 and Pro601. Based on the high level of purifying selection acting on this region and its location and orientation within the dimer structure, we suggest that it is a strong candidate to act as the receptor binding site 2. Mutagenesis experiments should be performed in order to investigate the magnitude of the possible individual contribution of these residues. Moreover, the contiguous residues Lys614, Trp615, Tp616, Pro617, Pro618, and Pro621 display a strict conservation pattern, and their possible role in binding or signal transduction should also be investigated further. In an attempt to validate this prediction, a search for naturally occurring or engineered mutations on these residues was carried out. However, no experimental evidence that could provide insight into the functional role of this surface was found reported in the RILM database [69] or in the NCBI SNPdb [81]. Furthermore, an evolutionary trace (ET) [82], [83], [84] run was performed for the IR MSA, as described in the Materials and Methods section. ET is an evolution-entropy hybrid method that assigns a relative score of functional importance to each sequence residue and subsequently ranks the residues by importance. Consistent with the likely acting high selective pressure, ET ranked first those hydrophobic residues located in the interior of the protein as well as some residues at the monomer-monomer and inter-domain interfaces (rho≈1.00), whereas residues comprising both the receptor binding site in L1 and the proposed binding site 2 scored moderately highly (rho≈1.74–3.00 and rho≈1–2.59, respectively), forming two uniformly conserved surfaces, as can be appreciated in Figure 5.

**Figure 6:**
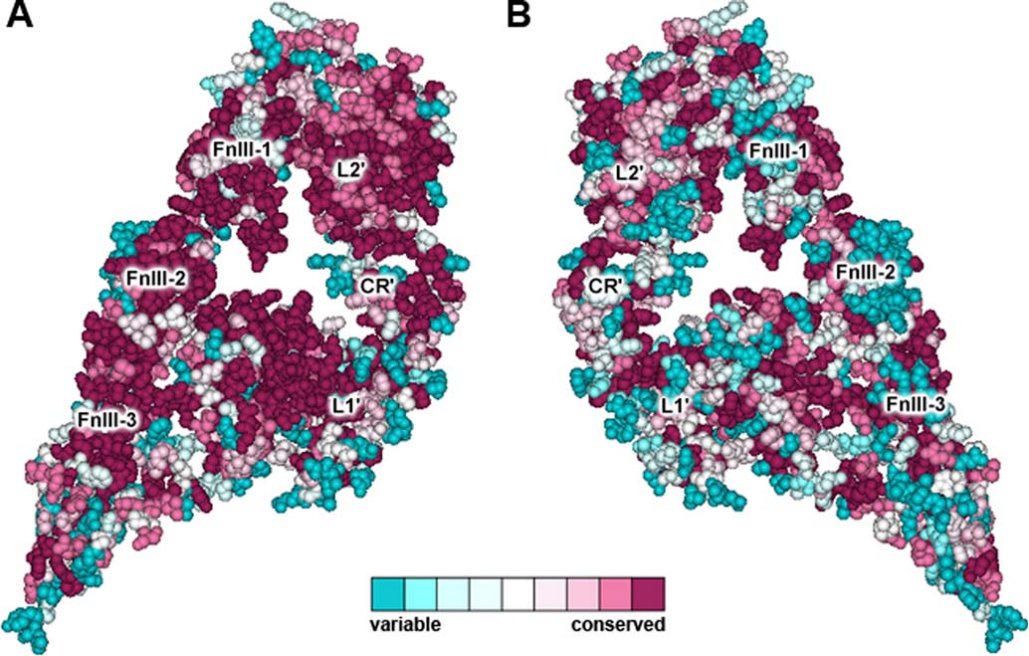
Conservation of a single insulin receptor binding site. Each IR features two binding sites. Each one of them is formed by two components: binding site 1 is contained in the first three domains of one monomer, and comprises the conserved surface on L1 and the carboxy-terminal of the ID, which could not be crystallised in the IR ectodomain structure. In IGF-1, binding additionally involves the CR region. Binding site 2 is contained in the other monomer and it is thought to involve one or more of the FnIII domains. This figure shows the conservation of the (A) inner and (B) outer surfaces of a single binding site. It is evident that the inner surface is considerably more conserved, due in part to its role in dimer formation. The figure is based on the coordinates of PDB structure 2DTG and residue conservation is indicated in the same colour scale as in previous figures.

**Figure 7:**
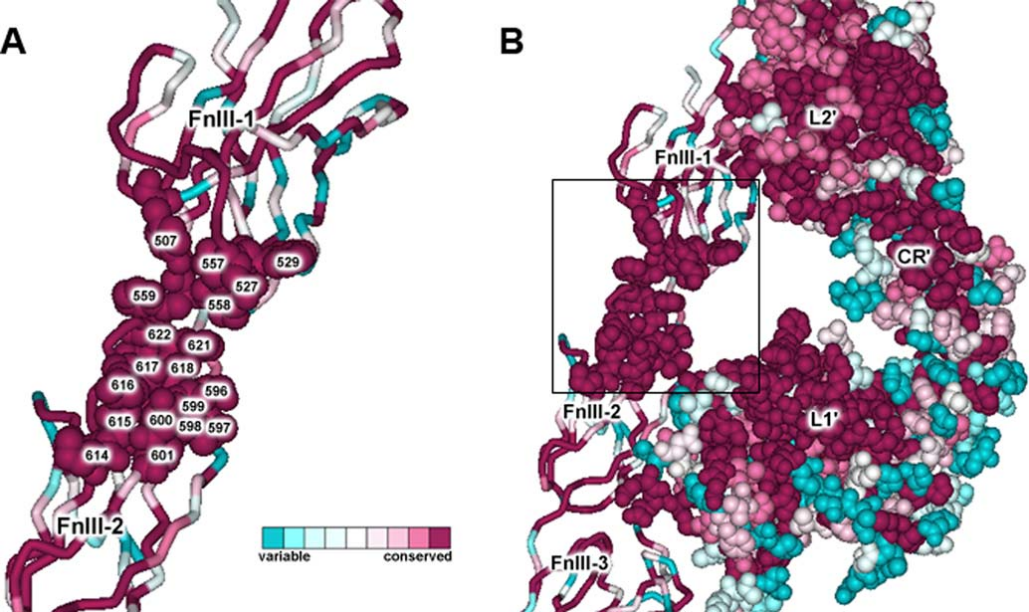
Proposed receptor second ligand binding site. (A) A region of conserved residues on the FnIII domains was identified and is proposed to act as the receptor binding site 2. Insulin is believed to cross-link both monomers in the high-affinity state of binding. Residues that are predicted to be involved in forming the receptor second binding site, and the corresponding residues at the same positions in the IGF1R and IRR, are listed in Table 4. (B) Representation of the location of the proposed binding site 2 within the dimer structure.

Figure 6 shows the conservation of a single dimer binding site, formed by the three FnIII domains from one monomer (FnIII-1-FnIII-2-FnIII-3) and the first three domains from the other monomer (L1'-CR'-L2') in the IR. It is evident once again that the inner surfaces are more conserved than the outer ones. The conserved residues that are likely to contact insulin upon binding are listed in Table 4 and their location in the structure of the IR is shown in Figure 7.

**Table 4:** Residues potentially involved in the second insulin binding site (numbering according to PDB structure 2DTG).

IR	IGF1R	IRR
Tyr507	Tyr	Tyr
Asn527	Asn	Gln
Trp529	Trp	Trp
Lys557	Lys	Lys
Pro558	Pro	Pro
Trp559	Trp	Trp
Ser596	Ser	Tre
Val597	Ile	Val
Pro598	Pro	Pro
Leu599	Leu	Gln
Asp600	Asp	Asp
Pro601	Val	Val
Lys614	Lys	Arg
Trp615	Trp	Trp
Lys616	Asn	Lys
Pro617	Pro	Pro
Pro618	Pro	Pro
Pro621	Pro	Arg

**Figure 8:**
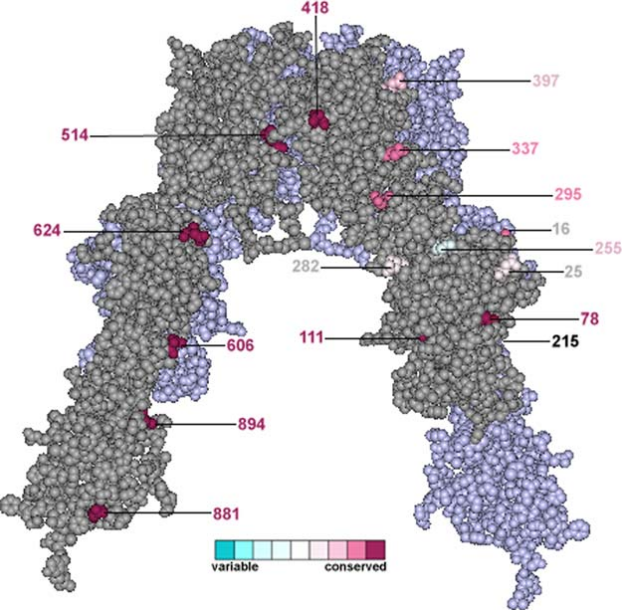
Conservation and location of the N-glycosylation sites of the IR. The figure shows the IR dimer structure. One monomer is displayed in gray and the other one in light purple. N-glycosylation sites are highlighted according to their conservation grade. Numbering is shown according to the 2DTG structure. Asn671, Asn730 and Asn743 lie within the un-crystallized ID.

The IR is a highly glycosylated protein, comprising both N- and O-linked glycosylation sites [85], [86]. The functions of the N-linked glycans attached to the IR include facilitating the correct folding of the protein, processing of the proreceptor and dimer formation, as well as the transport of the functional receptor to the membrane [4], [87], [88]. Studies aimed at investigating the effect of the removal of N-linked glycosylation sites suggest that there are redundancies in IR glycosylation, since many sites can be mutated individually without compromising cell surface expression, receptor processing or ligand binding [87]. On the other hand, when combinations of sites are mutated, folding cannot be carried out properly [87]. We looked at the conservation of the N-glycosylation sites and found that 9 out of 19 sites showed strict evolutionary conservation: Asn78, Asn111, Asn418, Asn514, Asn606, Asn624, Asn742, Asn881 and Asn894, while Asn295, Asn337, Asn671 and Asn743 showed a nearly strict pattern. Figure 8 shows the location of the N-glycosylation sites within the dimer structure. Surprisingly, several of these glycosylation sites were lost in IRR, such as Asn16, Asn111, Asn215, Asn255, Asn282, Asn337 and Asn418, which indicates that IRR has a different glycosylation pattern in comparison to IR, supporting the evidence of redundancy in the IR. Although the IR also contains mucin-type O-linked glycans attached to six Ser/Tre residues located in the N-terminal portion of the β chain, a recent study suggested that O-linked glycosylation is unlikely to be functionally significant in the IR family [89]. In the IGF1R there are only three O-linked glycosylations, whereas in the IRR there are only two serine residues in the corresponding portion and a single O-glycosylation site predicted [89].

Interestingly, we found a considerable number of conserved Gly and Pro residues located within the FnIII-1 and FnIII-2 domains. These residues may provide structural flexibility to this region, and may also play a specific role in preventing aggregation, as suggested by previous studies [90], [91].

From our results, it is clear that the L2 domain of the IR plays an important structural role in dimer formation through its interaction with the FnIII-1 domain from the other monomer, and that it contributes to the adoption of the “inverted V” conformation of the monomer through its interaction with the FnIII-1 domain from the same monomer. However, we did not find any conserved surface that could be involved in ligand binding. Furthermore, the contribution of the L2 domain to ligand binding in the IGF1R and IRR is still unclear.

Interestingly, in an attempt to develop insulin mimetic peptides, the use of phage display methodologies led to the discovery of three groups of peptides unrelated in sequence to insulin that recognise three different sites on the IR surface [92]. The synthetic combination of two of these sites resulted in a very potent, 36-residue single chain peptide with insulin mimetic activity that had an affinity for the IR comparable to that of insulin [93]. Further studies found that activation of the IR-A by this peptide, named S597, displays metabolic equipotency but low mitogenicity as compared to activation by insulin, supporting the idea that insulin and S597 elicit different signaling and biological responses through acting on the same IR isoform [94], later confirmed by gene expression profile analysis [95]. It is thus believed that there is more than one way to activate the IR.

Likewise, it has been reported that the soluble ectodomain of the IR shows only low-affinity ligand binding, unless it is tethered by transmembrane anchors, leucine zippers or Fc domains [32], [40]. It is also known that high affinity binding of insulin is accompanied by a structural compaction of its receptor [96]. It may therefore be necessary to consider that the conformation displayed by the IR in PDB structure 2DTG may not be the actual conformation of the receptor when insulin is bound to it.

Our residue conservation analysis reveals that, although a portion of the conserved surface on the FnIII domains points directly towards the conserved L1 surface, a slight rotation of the FnIII-1/FnIII-2 domains would be needed in order for the conserved surface to adopt an orientation that allows it to fully contact the insulin binding site 2. To test the likelihood of this conformational change, we performed a normal modes analysis (NMA) of a single IR monomer in order to predict its low frequency, high amplitude intrinsic vibrations. The results suggest that the receptor is prone to rotate the FnIII-2/FnIII-3 conserved surface towards the conserved surface on L1. On the other hand, this movement was not observed in the dimer structure. This is mainly explained by the restrictions imposed by inter-monomer interactions in the crystal structure of the ectodomain dimer, which may be different to the conformation that the receptor adopts when it is anchored to the cell membrane. A possible full rotation of the FnIII-2/FnIII-3 domains upon ligand binding could also act as a mechanical trigger for signal transduction in the tyrosine kinase domain of the IR. Further experiments and molecular dynamics simulations are needed to validate these predictions.

### Conclusions

Whilst the recent determination of the intact ectodomain structure of the IR by X-ray crystallography has provided new insights into the 3D arrangement of the receptor domains, a full understanding of its interactions with insulin and its functional activation remains elusive. The IR family thus remains a complex but interesting system of study, particularly as the physiological functions of heterodimeric receptors and the IRR are yet to be discovered.

The physiological role of the IR/IGF1R hetero-dimers is unknown and the physiological role of IRR is yet to be established. Our sequence analysis indicates that IRR is highly conserved throughout evolution, from *Xenopus laevis* to mammals, and that it differs from the IGF1R and IR in some key residues for ligand specificity.

In this study, amino acid residue conservation scores have revealed the different degrees of purifying selection acting on the protein surface of the IR and IGF1R. We have used this information to predict the location of the experimentally characterised ligand binding sites on the surfaces of L1 and CR as a control. These predictions were validated against the mutagenesis data available from the RILM online database [69], and were found to be in agreement with previous insulin binding models. No conserved surface on L2 was found pointing towards the receptor binding site. In addition, there does not appear to be any evidence that directly relates L2 to ligand binding.

A region of conserved residues on the surface of the FnIII domains was identified. Based on its location, this region is a strong candidate to act as the receptor insulin binding site 2. However, its location suggests the need for a slight ‘twist’ rotation of the FnIII-2/FnIII-3 domains with respect to FnIII-1 in order to face the likely location of insulin. This conformational change may act as a mechanical trigger for receptor activation and signal transduction. Further experiments and computer simulations are needed in order to validate these predictions.

The insulin binding model that proposes that insulin cross-links both receptor monomers in the IR also suggests that this is not needed for IGF-1 binding, which only requires binding site 1. This idea is supported by chimeric construct experiments that have shown that both IR-A/IGF1R and IR-B/IGF1R hybrids behave like the IGF1R.

Further crystallographic structures of both the low- and high-affinity ligand/receptor complexes for the IR and IGF1R are required to establish unambiguously the specific interactions involved in ligand binding and receptor structural components involved in these interactions, as well as to understand the nature of the structural transitions that lead to the activation of the receptor kinase. Due to the difficulties associated with the crystallisation of transmembrane receptors, mutagenesis data and molecular dynamics simulations may provide the easiest approaches to characterise the molecular basis of ligand binding and receptor activation.

Finally, this study demonstrates that methods that estimate amino acid sequence evolutionary conservation rates can provide valuable information about regions of functional importance upon the correct categorisation of homologous sequences into orthologous sets when crystal structures are available.

## Materials and Methods

### Data Sets

A BLAST (tblastn) [97] search was performed against the GenBank non-redundant nucleotide database [98] and the ENSEMBL nucleotide database [99], using NCBI and ENSEMBL web site tools [99], [100]. The query sequences corresponded to those of the IR family in humans: AAA59174.1 (IR), AAB22215 (IGF1R) and NP_055030 (IRR). Homologous sequences were subsequently classified into three different sets (IR, IGF1R and IRR) of orthologous sequences. Orthology was validated by a bi-directional best hit procedure [101]. The sequences and their accession numbers in the final sets for the IR, IGF1R and IRR are listed in Table 1.

Additional modifications were made to the following sequences: Bos Taurus IR mRNA was found to be reported in three separate but overlapping transcripts and was merged manually by removing the overlapping regions. Similarly, two different but complementary mRNAs were found for *Pan Troglodytes*, and were merged into a single sequence. Masked residues were removed from *Echinops Telfairi* and *Myotis Lucifugus* IR and from *Erinaceus Europeus* IGF1R sequences. The original and edited sequences can be provided upon request to the authors.

### Phylogenetic Analysis of the IR family

The three sets (IR, IGF1R and IRR) of orthologous sequences listed in Table 1, and that corresponding to the IR family receptor ectodomain of *Bombyx Mori* (Silkworm) [NP_001037011], *Drosophila Melanogaster* (Fruit fly) [NP_524436.2] and *Lymnaea stagnalis* (Great pond snail) [CAA59353], were aligned separately using MUSCLE [62] with three refinement rounds. The four sets of Multiple Sequence Alignments (MSA) were finally merged into a single one by profile to profile alignment using MUSCLE. All the alignments and their respective phylogenetic trees used in this study are provided in Supplementary File S3.

### IR Family Phylogenetic Analysis

Bayesian (By) and maximum likelihood (ML) tree searches were conducted using the MSAs produced by MUSCLE of the IR family ectodomain, residues N-terminal from His1 or C-terminal from Leu909 (human IR numbering as in the crystal structure) were not taken into account in the alignments. ML tree searches were performed with PhyML 2.4.5 [102], [103] for each of the alignment sets (IR, IGF1R and IRR) under the best-approximating model selected by ProtTest [63] using the Akaike information criterion [104]. A ML tree search was also performed for the full data set (including the 3 invertebrate sequences) under the model with the highest posterior probability found by MrBayes, as explained below. In order to make a more thorough search of tree space for the full dataset, 40 random step-wise addition parsimony trees were generated with PAUP*4b10 [105] and used to initiate a corresponding number of ML searches on a cluster of 27 dual core Pentium IV processors under Linux Rocks 3.3.0. A default PhyML search using a BioNJ seed tree was also used. The tree yielding the highest log-likelihood (ln*L*) value was selected amongst the 41 independent searches. The robustness of the ML topologies was evaluated using a recently developed Shimodaira-Hasegawa-like test for branches [103] implemented in PhyML v2.4.5 [102]. In brief, the test assesses whether the branch being studied provides a significant likelihood gain, in comparison with the null hypothesis that involves collapsing that branch, but leaving the rest of the tree topology identical. We chose the Shimodaira-Hasegawa-like procedure for assessing bipartition significance because the test is non-parametric and much less liberal than the diverse (parametric) approximate likelihood ratio tests (aLRTs) that are also implemented in that program [103]. The resulting SH-like *P*-values therefore indicate the probability that the corresponding split is significant. A Bayesian estimation of phylogeny was performed with MrBayes 3.1.2 [106] for the full dataset. Two independent Metropolis-coupled Markov-chain Monte Carlo (MC^3^) simulations were run for 5×10^5^ generations, sampling every 100^th^, using three heated chains (temperature parameter set to 0.2) and a reversible-jump model prior to use the chain for model selection. Each independent MC^3^ run had two replicates and was requested to use gamma-distributed rates. The first 1000 samples (20%) were discarded as burnin. Convergence and proper mixing of the chains was evaluated by visual inspection of generation plots, comparison of the arithmetic and harmonic ln*L* means of the two replicate runs, and calculation of symmetric Robinson-Fould tree distances within replicates of the same run (using the MrBayes sump output) and Treedist of the Phylip package [107] to compute these distances between the majority rule consensus trees obtained from each independent MC^3^ run (all provided in Supplementary File S1 and Supplementary File S2).

The overall resolution of Bayesian and ML trees was evaluated by computing diverse descriptive statistics of the SH-like *P* values or Bayesian posterior probabilities parsed from the corresponding phylograms using *ad hoc* Perl scripts [8].

### Calculation of Consurf Conservation Scores

The conservation scores at each amino acid position were calculated with the Rate4Site algorithm [53], under the maximum likelihood (ML) principle providing both the IGF1R and the IR MSAs and their corresponding ML trees calculated as explained above. The conservation scores were projected onto the crystal structures of the IR ectodomain (PDB code 2DTG), the IR first three domains (PDB code 2HR7) and the IGF1R first three domains (PDB code 1IGR), after submitting the data to the ConSurf server [68], [104]. Consurf results for both the IR and the IGF1R sets are provided in Supplementary File S4.

### Evolutionary Trace Calculation

Evolutionary Trace calculations were performed by running the ET Wizard module coupled into the Evolutionary Trace Viewer 2.0 remotely, providing the IR MSA and the 2PDB E chain as input. Complete ET results are provided in Supplementary File S5.

### Normal Modes Analysis

Normal modes analysis (NMA) was used to predict the equilibrium low frequency, high amplitude inter-domain movements of an IR monomer, using the Elastic Network Model (ENM) as available through the *ElNémo* web server [108]. PDB structure 2DTG was used as input for these calculations. The five lowest frequency normal modes were computed and the minimum and maximum perturbations were set to −150 and 150 DQ, respectively. The output in PDB format corresponding to the fourth model is provided in Supplementary File S6.

## Supporting Information

Supplementary File S1 Supplemental Information for the Maximum-likelihood Analyses
(0.10 MB PDF)

Supplementary File S2 Supplemental Information for the Bayesian Analyses
(0.65 MB PDF)

Supplementary File S3 Zip file containing all multiple sequence alignments and phylogenetic trees used in this study
(0.03 MB ZIP)

Supplementary File S4 Zip file containing Consurf scores for both the IR and the IGF1R ectodomains
(0.02 MB ZIP)

Supplementary File S5 Zip file containing Evolutionary Trace results for the IR
(0.75 MB ZIP)

Supplementary File S6 Zip file containing PDB structures obtained through normal modes calculations.
(1.20 MB ZIP)
