# Supplementary material for: A Comparative Structural Bioinformatics Analysis of the Insulin Receptor Family Ectodomain Based on Phylogenetic Information
Source: PLoS One. 2008 Nov 7;3(11):e3667. doi: 10.1371/journal.pone.0003667 (PMC2577065; doi:10.1371/journal.pone.0003667)
Supplement: Supplementary File S1 — Supplemental Information for the Maximum-likelihood Analyses (0.10 MB PDF) [file pone.0003667.s001.pdf]

# Supplemental data for the ML analyses

## A) ProtTest model selection for the three sets of Vertebrate IR homologues.

### 1) Model selection for the IGF1R set of orthologues.

************************************************************
**Best model according to AIC: JTT+G**
************************************************************

| Model | deltaAIC* | AIC | AICw | -lnL |
|---|---|---|---|---|
| JTT+G | 0.00 | 18858.28 | **0.73** | -9391.14 |
| JTT+I+G | 2.01 | 18860.28 | 0.27 | -9391.14 |
| WAG+G | 145.41 | 19003.69 | 0.00 | -9463.85 |
| WAG+I+G | 147.42 | 19005.70 | 0.00 | -9463.85 |
| JTT+I | 191.61 | 19049.89 | 0.00 | -9486.94 |
| Dayhoff+G | 237.47 | 19095.75 | 0.00 | -9509.87 |
| Dayhoff+I+G | 239.47 | 19097.75 | 0.00 | -9509.88 |
| WAG+I | 313.48 | 19171.76 | 0.00 | -9547.88 |
| Blosum62+G | 384.49 | 19242.77 | 0.00 | -9583.39 |
| Blosum62+I+G | 386.50 | 19244.78 | 0.00 | -9583.39 |
| VT+G | 393.59 | 19251.87 | 0.00 | -9587.94 |
| VT+I+G | 395.60 | 19253.88 | 0.00 | -9587.94 |
| Dayhoff+I | 429.91 | 19288.19 | 0.00 | -9606.09 |
| JTT | 472.33 | 19330.61 | 0.00 | -9628.31 |
| Blosum62+I | 560.18 | 19418.46 | 0.00 | -9671.23 |
| WAG | 578.90 | 19437.18 | 0.00 | -9681.59 |
| VT+I | 645.22 | 19503.50 | 0.00 | -9713.75 |
| Dayhoff | 727.97 | 19586.25 | 0.00 | -9756.13 |
| Blosum62 | 816.85 | 19675.13 | 0.00 | -9800.57 |
| VT | 950.02 | 19808.30 | 0.00 | -9867.15 |

*: models sorted according to this column

************************************************
Relative importance of parameters
************************************************

| | | |
|---|---|---|
| **alpha** | **(+G):** | **0.73** |
| p-inv | (+I): | 0.00 |
| alpha+p-inv | (+I+G): | 0.27 |
| freqs | (+F): | 0.00 |

************************************************
Model-averaged estimate of parameters
************************************************

| | |
|---|---|
| alpha (+G): | 0.69 |
| p-inv (+I): | 0.30 |
| alpha (+I+G): | 0.69 |
| p-inv (+I+G): | 0.00 |

Table: Weights(Ranking) of the candidate models under the different frameworks

| model | AIC | AICc-1 | AICc-2 | AICc-3 | BIC-1 | BIC-2 | BIC-3 |
|---|---|---|---|---|---|---|---|
| **JTT+G** | **0.73(1)** | **0.75(1)** | **0.77(1)** | **0.74(1)** | **0.97(1)** | **0.95(1)** | **0.98(1)** |
| JTT+I+G | 0.27(2) | 0.25(2) | 0.23(2) | 0.26(2) | 0.03(2) | 0.05(2) | 0.02(2) |
| WAG+G | 0.00(3) | 0.00(3) | 0.00(3) | 0.00(3) | 0.00(3) | 0.00(3) | 0.00(3) |
| WAG+I+G | 0.00(4) | 0.00(4) | 0.00(4) | 0.00(4) | 0.00(4) | 0.00(4) | 0.00(4) |
| JTT+I | 0.00(5) | 0.00(5) | 0.00(5) | 0.00(5) | 0.00(5) | 0.00(5) | 0.00(5) |
| Dayhoff+G | 0.00(6) | 0.00(6) | 0.00(6) | 0.00(6) | 0.00(6) | 0.00(6) | 0.00(6) |
| Dayhoff+I+G | 0.00(7) | 0.00(7) | 0.00(7) | 0.00(7) | 0.00(7) | 0.00(7) | 0.00(7) |
| WAG+I | 0.00(8) | 0.00(8) | 0.00(8) | 0.00(8) | 0.00(8) | 0.00(8) | 0.00(8) |
| Blosum62+G | 0.00(9) | 0.00(9) | 0.00(9) | 0.00(9) | 0.00(9) | 0.00(9) | 0.00(9) |
| Blosum62+I+G | 0.00(10) | 0.00(10) | 0.00(10) | 0.00(10) | 0.00(10) | 0.00(10) | 0.00(10) |
| VT+G | 0.00(11) | 0.00(11) | 0.00(11) | 0.00(11) | 0.00(11) | 0.00(11) | 0.00(11) |
| VT+I+G | 0.00(12) | 0.00(12) | 0.00(12) | 0.00(12) | 0.00(12) | 0.00(12) | 0.00(12) |
| Dayhoff+I | 0.00(13) | 0.00(13) | 0.00(13) | 0.00(13) | 0.00(13) | 0.00(13) | 0.00(13) |
| JTT | 0.00(14) | 0.00(14) | 0.00(14) | 0.00(14) | 0.00(14) | 0.00(14) | 0.00(14) |
| Blosum62+I | 0.00(15) | 0.00(15) | 0.00(15) | 0.00(15) | 0.00(15) | 0.00(15) | 0.00(15) |
| WAG | 0.00(16) | 0.00(16) | 0.00(16) | 0.00(16) | 0.00(16) | 0.00(16) | 0.00(16) |
| VT+I | 0.00(17) | 0.00(17) | 0.00(17) | 0.00(17) | 0.00(17) | 0.00(17) | 0.00(17) |
| Dayhoff | 0.00(18) | 0.00(18) | 0.00(18) | 0.00(18) | 0.00(18) | 0.00(18) | 0.00(18) |
| Blosum62 | 0.00(19) | 0.00(19) | 0.00(19) | 0.00(19) | 0.00(19) | 0.00(19) | 0.00(19) |
| VT | 0.00(20) | 0.00(20) | 0.00(20) | 0.00(20) | 0.00(20) | 0.00(20) | 0.00(20) |

| Relative importance of parameters | AIC | AICc-1 | AICc-2 | AICc-3 | BIC-1 | BIC-2 | BIC-3 |
|---|---|---|---|---|---|---|---|
| **+G** | **0.73** | **0.75** | **0.77** | **0.74** | **0.97** | **0.95** | **0.98** |
| +I | 0.00 | 0.00 | 0.00 | 0.00 | 0.00 | 0.00 | 0.00 |
| +I+G | 0.27 | 0.25 | 0.23 | 0.26 | 0.03 | 0.05 | 0.02 |
| +F | 0.00 | 0.00 | 0.00 | 0.00 | 0.00 | 0.00 | 0.00 |

| Model-averaged estimate of parameters | AIC | AICc-1 | AICc-2 | AICc-3 | BIC-1 | BIC-2 | BIC-3 |
|---|---|---|---|---|---|---|---|
| alpha (+G) | 0.69 | 0.69 | 0.69 | 0.69 | 0.69 | 0.69 | 0.69 |
| p-inv (+I) | 0.30 | 0.30 | 0.30 | 0.30 | 0.30 | 0.30 | 0.30 |
| alpha (+I+G) | 0.69 | 0.69 | 0.69 | 0.69 | 0.69 | 0.69 | 0.69 |
| p-inv (+I+G) | 0.00 | 0.00 | 0.00 | 0.00 | 0.00 | 0.00 | 0.00 |

AIC : Akaike Information Criterion framework.
AICc-x: Second-Order Akaike framework.
BIC-x : Bayesian Information Criterion framework.
AICc/BIC-1: sample size as: number of sites in the alignment (946.0)
AICc/BIC-2: sample size as: Sum of position's Shannon Entropy over the whole alignment (441.7)
AICc/BIC-3: sample size as: align. length x num sequences x averaged (0-1)Sh. Entropy (2043.8)

## 2) Model selection for the IR set of orthologues.

**Best model according to AIC: JTT+G**

| Model | deltaAIC* | AIC | AICw | -lnL |
|---|---|---|---|---|
| JTT+G | 0.00 | 21501.00 | **0.72** | -10712.50 |
| JTT+I+G | 1.87 | 21502.88 | 0.28 | -10712.44 |
| WAG+G | 163.79 | 21664.79 | 0.00 | -10794.40 |
| WAG+I+G | 165.40 | 21666.40 | 0.00 | -10794.20 |
| Dayhoff+G | 228.00 | 21729.00 | 0.00 | -10826.50 |
| Dayhoff+I+G | 229.96 | 21730.96 | 0.00 | -10826.48 |
| JTT+I | 302.29 | 21803.29 | 0.00 | -10863.65 |
| VT+G | 439.22 | 21940.22 | 0.00 | -10932.11 |
| VT+I+G | 441.22 | 21942.22 | 0.00 | -10932.11 |
| WAG+I | 449.45 | 21950.45 | 0.00 | -10937.23 |
| Blosum62+G | 455.08 | 21956.09 | 0.00 | -10940.04 |
| Blosum62+I+G | 456.89 | 21957.90 | 0.00 | -10939.95 |
| Dayhoff+I | 559.59 | 22060.59 | 0.00 | -10992.30 |
| Blosum62+I | 728.09 | 22229.10 | 0.00 | -11076.55 |
| JTT | 771.96 | 22272.96 | 0.00 | -11099.48 |
| VT+I | 814.43 | 22315.44 | 0.00 | -11119.72 |
| WAG | 896.02 | 22397.03 | 0.00 | -11161.51 |
| Dayhoff | 1032.70 | 22533.70 | 0.00 | -11229.85 |
| Blosum62 | 1175.88 | 22676.88 | 0.00 | -11301.44 |
| VT | 1301.25 | 22802.26 | 0.00 | -11364.13 |

*: models sorted according to this column

Relative importance of parameters

| | | |
|---|---|---|
| **alpha** | **(+G):** | **0.72** |
| p-inv | (+I): | 0.00 |
| alpha+p-inv | (+I+G): | 0.28 |
| freqs | (+F): | 0.00 |

Model-averaged estimate of parameters

| | |
|---|---|
| alpha (+G): | 0.57 |
| p-inv (+I): | 0.33 |
| alpha (+I+G): | 0.60 |
| p-inv (+I+G): | 0.02 |

Table: Weights(Ranking) of the candidate models under the different frameworks

| model | AIC | AICc-1 | AICc-2 | AICc-3 | BIC-1 | BIC-2 | BIC-3 |
|---|---|---|---|---|---|---|---|
| **JTT+G** | **0.72(1)** | **0.74(1)** | **0.75(1)** | **0.73(1)** | **0.97(1)** | **0.95(1)** | **0.98(1)** |
| JTT+I+G | 0.28(2) | 0.26(2) | 0.25(2) | 0.27(2) | 0.03(2) | 0.05(2) | 0.02(2) |
| WAG+G | 0.00(3) | 0.00(3) | 0.00(3) | 0.00(3) | 0.00(3) | 0.00(3) | 0.00(3) |
| WAG+I+G | 0.00(4) | 0.00(4) | 0.00(4) | 0.00(4) | 0.00(4) | 0.00(4) | 0.00(4) |
| Dayhoff+G | 0.00(5) | 0.00(5) | 0.00(5) | 0.00(5) | 0.00(5) | 0.00(5) | 0.00(5) |
| Dayhoff+I+G | 0.00(6) | 0.00(6) | 0.00(6) | 0.00(6) | 0.00(6) | 0.00(6) | 0.00(6) |
| JTT+I | 0.00(7) | 0.00(7) | 0.00(7) | 0.00(7) | 0.00(7) | 0.00(7) | 0.00(7) |
| VT+G | 0.00(8) | 0.00(8) | 0.00(8) | 0.00(8) | 0.00(8) | 0.00(8) | 0.00(8) |
| VT+I+G | 0.00(9) | 0.00(9) | 0.00(9) | 0.00(9) | 0.00(9) | 0.00(9) | 0.00(9) |
| WAG+I | 0.00(10) | 0.00(10) | 0.00(10) | 0.00(10) | 0.00(10) | 0.00(10) | 0.00(10) |
| Blosum62+G | 0.00(11) | 0.00(11) | 0.00(11) | 0.00(11) | 0.00(11) | 0.00(11) | 0.00(11) |
| Blosum62+I+G | 0.00(12) | 0.00(12) | 0.00(12) | 0.00(12) | 0.00(12) | 0.00(12) | 0.00(12) |
| Dayhoff+I | 0.00(13) | 0.00(13) | 0.00(13) | 0.00(13) | 0.00(13) | 0.00(13) | 0.00(13) |
| Blosum62+I | 0.00(14) | 0.00(14) | 0.00(14) | 0.00(14) | 0.00(14) | 0.00(14) | 0.00(14) |
| JTT | 0.00(15) | 0.00(15) | 0.00(15) | 0.00(15) | 0.00(15) | 0.00(15) | 0.00(15) |
| VT+I | 0.00(16) | 0.00(16) | 0.00(16) | 0.00(16) | 0.00(16) | 0.00(16) | 0.00(16) |
| WAG | 0.00(17) | 0.00(17) | 0.00(17) | 0.00(17) | 0.00(17) | 0.00(17) | 0.00(17) |
| Dayhoff | 0.00(18) | 0.00(18) | 0.00(18) | 0.00(18) | 0.00(18) | 0.00(18) | 0.00(18) |
| Blosum62 | 0.00(19) | 0.00(19) | 0.00(19) | 0.00(19) | 0.00(19) | 0.00(19) | 0.00(19) |
| VT | 0.00(20) | 0.00(20) | 0.00(20) | 0.00(20) | 0.00(20) | 0.00(20) | 0.00(20) |

| Relative importance of parameters | AIC | AICc-1 | AICc-2 | AICc-3 | BIC-1 | BIC-2 | BIC-3 |
|---|---|---|---|---|---|---|---|
| **+G** | **0.72** | **0.74** | **0.75** | **0.73** | **0.97** | **0.95** | **0.98** |
| +I | 0.00 | 0.00 | 0.00 | 0.00 | 0.00 | 0.00 | 0.00 |
| +I+G | 0.28 | 0.26 | 0.25 | 0.27 | 0.03 | 0.05 | 0.02 |
| +F | 0.00 | 0.00 | 0.00 | 0.00 | 0.00 | 0.00 | 0.00 |

| Model-averaged estimate of parameters | AIC | AICc-1 | AICc-2 | AICc-3 | BIC-1 | BIC-2 | BIC-3 |
|---|---|---|---|---|---|---|---|
| alpha (+G) | 0.57 | 0.57 | 0.57 | 0.57 | 0.57 | 0.57 | 0.57 |
| p-inv (+I) | 0.33 | 0.33 | 0.33 | 0.33 | 0.33 | 0.33 | 0.33 |
| alpha (+I+G) | 0.60 | 0.60 | 0.60 | 0.60 | 0.60 | 0.60 | 0.60 |
| p-inv (+I+G) | 0.02 | 0.02 | 0.02 | 0.02 | 0.02 | 0.02 | 0.02 |

AIC : Akaike Information Criterion framework.
AICc-x: Second-Order Akaike framework.
BIC-x : Bayesian Information Criterion framework.
AICc/BIC-1: sample size as: number of sites in the alignment (946.0)
AICc/BIC-2: sample size as: Sum of position's Shannon Entropy over the whole alignment (490.4)
AICc/BIC-3: sample size as: align. length x num sequences x averaged (0-1)Sh. Entropy (2269.5)

## 3) Model selection for the IRR set of orthologues.

************************************************************
**Best model according to AIC: JTT+G**
************************************************************

| Model | deltaAIC* | AIC | AICw | -lnL |
|---|---|---|---|---|
| JTT+G | 0.00 | 14108.65 | **0.73** | -7026.33 |
| JTT+I+G | 2.00 | 14110.65 | 0.27 | -7026.33 |
| JTT+I | 109.39 | 14218.05 | 0.00 | -7081.02 |
| WAG+G | 186.18 | 14294.84 | 0.00 | -7119.42 |
| WAG+I+G | 188.18 | 14296.84 | 0.00 | -7119.42 |
| Dayhoff+G | 218.53 | 14327.18 | 0.00 | -7135.59 |
| Dayhoff+I+G | 220.53 | 14329.18 | 0.00 | -7135.59 |
| WAG+I | 309.21 | 14417.86 | 0.00 | -7180.93 |
| JTT | 345.54 | 14454.19 | 0.00 | -7200.09 |
| Dayhoff+I | 345.69 | 14454.35 | 0.00 | -7199.17 |
| VT+G | 401.38 | 14510.03 | 0.00 | -7227.01 |
| VT+I+G | 403.38 | 14512.03 | 0.00 | -7227.01 |
| Blosum62+G | 422.83 | 14531.48 | 0.00 | -7237.74 |
| Blosum62+I+G | 424.83 | 14533.48 | 0.00 | -7237.74 |
| WAG | 538.72 | 14647.37 | 0.00 | -7296.69 |
| VT+I | 542.73 | 14651.38 | 0.00 | -7297.69 |
| Blosum62+I | 545.11 | 14653.77 | 0.00 | -7298.88 |
| Dayhoff | 604.66 | 14713.32 | 0.00 | -7329.66 |
| Blosum62 | 767.41 | 14876.06 | 0.00 | -7411.03 |
| VT | 796.95 | 14905.61 | 0.00 | -7425.80 |

*: models sorted according to this column

************************************************
Relative importance of parameters
************************************************

| | | |
|---|---|---|
| **alpha** | **(+G):** | **0.73** |
| p-inv | (+I): | 0.00 |
| alpha+p-inv | (+I+G): | 0.27 |
| freqs | (+F): | 0.00 |

************************************************
Model-averaged estimate of parameters
************************************************

| | |
|---|---|
| alpha (+G): | 0.51 |
| p-inv (+I): | 0.42 |
| alpha (+I+G): | 0.51 |
| p-inv (+I+G): | 0.00 |

Table: Weights(Ranking) of the candidate models under the different frameworks

| model | AIC | AICc-1 | AICc-2 | AICc-3 | BIC-1 | BIC-2 | BIC-3 |
|---|---|---|---|---|---|---|---|
| **JTT+G** | **0.73(1)** | **0.74(1)** | **0.77(1)** | **0.74(1)** | **0.97(1)** | **0.95(1)** | **0.97(1)** |
| JTT+I+G | 0.27(2) | 0.26(2) | 0.23(2) | 0.26(2) | 0.03(2) | 0.05(2) | 0.03(2) |
| JTT+I | 0.00(3) | 0.00(3) | 0.00(3) | 0.00(3) | 0.00(3) | 0.00(3) | 0.00(3) |
| WAG+G | 0.00(4) | 0.00(4) | 0.00(4) | 0.00(4) | 0.00(4) | 0.00(4) | 0.00(4) |
| WAG+I+G | 0.00(5) | 0.00(5) | 0.00(5) | 0.00(5) | 0.00(5) | 0.00(5) | 0.00(5) |
| Dayhoff+G | 0.00(6) | 0.00(6) | 0.00(6) | 0.00(6) | 0.00(6) | 0.00(6) | 0.00(6) |
| Dayhoff+I+G | 0.00(7) | 0.00(7) | 0.00(7) | 0.00(7) | 0.00(7) | 0.00(7) | 0.00(7) |
| WAG+I | 0.00(8) | 0.00(8) | 0.00(8) | 0.00(8) | 0.00(8) | 0.00(8) | 0.00(8) |
| JTT | 0.00(9) | 0.00(9) | 0.00(9) | 0.00(9) | 0.00(9) | 0.00(9) | 0.00(9) |
| Dayhoff+I | 0.00(10) | 0.00(10) | 0.00(10) | 0.00(10) | 0.00(10) | 0.00(10) | 0.00(10) |
| VT+G | 0.00(11) | 0.00(11) | 0.00(11) | 0.00(11) | 0.00(11) | 0.00(11) | 0.00(11) |
| VT+I+G | 0.00(12) | 0.00(12) | 0.00(12) | 0.00(12) | 0.00(12) | 0.00(12) | 0.00(12) |
| Blosum62+G | 0.00(13) | 0.00(13) | 0.00(13) | 0.00(13) | 0.00(13) | 0.00(13) | 0.00(13) |
| Blosum62+I+G | 0.00(14) | 0.00(14) | 0.00(14) | 0.00(14) | 0.00(14) | 0.00(14) | 0.00(14) |
| WAG | 0.00(15) | 0.00(15) | 0.00(15) | 0.00(15) | 0.00(15) | 0.00(15) | 0.00(15) |
| VT+I | 0.00(16) | 0.00(16) | 0.00(16) | 0.00(16) | 0.00(16) | 0.00(16) | 0.00(16) |
| Blosum62+I | 0.00(17) | 0.00(17) | 0.00(17) | 0.00(17) | 0.00(17) | 0.00(17) | 0.00(17) |
| Dayhoff | 0.00(18) | 0.00(18) | 0.00(18) | 0.00(18) | 0.00(18) | 0.00(18) | 0.00(18) |
| Blosum62 | 0.00(19) | 0.00(19) | 0.00(19) | 0.00(19) | 0.00(19) | 0.00(19) | 0.00(19) |
| VT | 0.00(20) | 0.00(20) | 0.00(20) | 0.00(20) | 0.00(20) | 0.00(20) | 0.00(20) |

| Relative importance of parameters | AIC | AICc-1 | AICc-2 | AICc-3 | BIC-1 | BIC-2 | BIC-3 |
|---|---|---|---|---|---|---|---|
| **+G** | **0.73** | **0.74** | **0.77** | **0.74** | **0.97** | **0.95** | **0.97** |
| +I | 0.00 | 0.00 | 0.00 | 0.00 | 0.00 | 0.00 | 0.00 |
| +I+G | 0.27 | 0.26 | 0.23 | 0.26 | 0.03 | 0.05 | 0.03 |
| +F | 0.00 | 0.00 | 0.00 | 0.00 | 0.00 | 0.00 | 0.00 |

| Model-averaged estimate of parameters | AIC | AICc-1 | AICc-2 | AICc-3 | BIC-1 | BIC-2 | BIC-3 |
|---|---|---|---|---|---|---|---|
| alpha (+G) | 0.51 | 0.51 | 0.51 | 0.51 | 0.51 | 0.51 | 0.51 |
| p-inv (+I) | 0.42 | 0.42 | 0.42 | 0.42 | 0.42 | 0.42 | 0.42 |
| alpha (+I+G) | 0.51 | 0.51 | 0.51 | 0.51 | 0.51 | 0.51 | 0.51 |
| p-inv (+I+G) | 0.00 | 0.00 | 0.00 | 0.00 | 0.00 | 0.00 | 0.00 |

AIC : Akaike Information Criterion framework.
AICc-x: Second-Order Akaike framework.
BIC-x : Bayesian Information Criterion framework.
AICc/BIC-1: sample size as: number of sites in the alignment (920.0)
AICc/BIC-2: sample size as: Sum of position's Shannon Entropy over the whole alignment (295.2)
AICc/BIC-3: sample size as: align. length x num sequences x averaged (0-1)Sh. Entropy (1024.5)

## B) Tree bipartition support statistics.

Based on the Shimodaira-Hasegawa-like *P* values for branch support calculated for each of the trees found in 41 PhyML maximum likelihood searches under JTT+G (40 using random sequential addition seed trees, the remaining one using a BioNJ tree).

| tree_file | n_bip. | min | max | mean | median | std_dev | variance | %_NRB < 0.7 | %_PRB [ 0.7-.85) | %_MRB [ .85-.95) | %_WRB [ .95-.99) | %_HRB ≥ 0.99 |
|---|---|---|---|---|---|---|---|---|---|---|---|---|
| my_IRR_IGF1R_IR_Invert_prof,phy10 | 55 | 0,01 | 1 | 0,82 | 0,94 | 0,26 | 0,0657 | 18,18 | 14,55 | 25,45 | 14,55 | 27,27 |
| my_IRR_IGF1R_IR_Invert_prof,phy11 | 55 | 0,08 | 1 | 0,83 | 0,94 | 0,25 | 0,0635 | 16,36 | 14,55 | 21,82 | 18,18 | 29,09 |
| my_IRR_IGF1R_IR_Invert_prof,phy12 | 55 | 0,21 | 1 | 0,83 | 0,94 | 0,23 | 0,0529 | 21,82 | 9,09 | 23,64 | 16,36 | 29,09 |
| my_IRR_IGF1R_IR_Invert_prof,phy13 | 55 | 0,1 | 1 | 0,84 | 0,94 | 0,23 | 0,0521 | 16,36 | 16,36 | 18,18 | 20 | 29,09 |
| my_IRR_IGF1R_IR_Invert_prof,phy14 | 55 | 0,01 | 1 | 0,8 | 0,92 | 0,28 | 0,0797 | 20 | 14,55 | 23,64 | 14,55 | 27,27 |
| my_IRR_IGF1R_IR_Invert_prof,phy15 | 55 | 0,17 | 1 | 0,82 | 0,92 | 0,24 | 0,0561 | 23,64 | 9,09 | 23,64 | 14,55 | 29,09 |
| my_IRR_IGF1R_IR_Invert_prof,phy16 | 55 | 0,06 | 1 | 0,85 | 0,94 | 0,21 | 0,0423 | 12,73 | 21,82 | 18,18 | 18,18 | 29,09 |
| my_IRR_IGF1R_IR_Invert_prof,phy17 | 55 | 0,01 | 1 | 0,83 | 0,93 | 0,24 | 0,0582 | 18,18 | 18,18 | 18,18 | 18,18 | 27,27 |
| my_IRR_IGF1R_IR_Invert_prof,phy18 | 55 | 0,01 | 1 | 0,83 | 0,93 | 0,23 | 0,0513 | 20 | 18,18 | 16,36 | 20 | 25,45 |
| my_IRR_IGF1R_IR_Invert_prof,phy19 | 55 | 0,29 | 1 | 0,87 | 0,93 | 0,17 | 0,0279 | 16,36 | 14,55 | 25,45 | 14,55 | 29,09 |
| my_IRR_IGF1R_IR_Invert_prof,phy1 | 55 | 0,01 | 1 | 0,79 | 0,94 | 0,28 | 0,0807 | 25,45 | 12,73 | 18,18 | 14,55 | 29,09 |
| my_IRR_IGF1R_IR_Invert_prof,phy20 | 55 | 0,05 | 1 | 0,82 | 0,94 | 0,25 | 0,0638 | 20 | 14,55 | 16,36 | 18,18 | 30,91 |
| my_IRR_IGF1R_IR_Invert_prof,phy21 | 55 | 0,02 | 1 | 0,84 | 0,93 | 0,23 | 0,0525 | 12,73 | 20 | 23,64 | 16,36 | 27,27 |
| my_IRR_IGF1R_IR_Invert_prof,phy22 | 55 | 0,26 | 1 | 0,83 | 0,93 | 0,21 | 0,0422 | 23,64 | 14,55 | 20 | 14,55 | 27,27 |
| my_IRR_IGF1R_IR_Invert_prof,phy23 | 55 | 0 | 1 | 0,81 | 0,94 | 0,27 | 0,0713 | 20 | 14,55 | 20 | 18,18 | 27,27 |
| my_IRR_IGF1R_IR_Invert_prof,phy24 | 55 | 0,01 | 1 | 0,81 | 0,93 | 0,28 | 0,0796 | 18,18 | 10,91 | 29,09 | 16,36 | 25,45 |
| my_IRR_IGF1R_IR_Invert_prof,phy25 | 55 | 0,01 | 1 | 0,81 | 0,94 | 0,27 | 0,071 | 20 | 12,73 | 20 | 16,36 | 30,91 |
| my_IRR_IGF1R_IR_Invert_prof,phy26 | 55 | 0,02 | 1 | 0,81 | 0,93 | 0,26 | 0,069 | 23,64 | 12,73 | 20 | 18,18 | 25,45 |
| my_IRR_IGF1R_IR_Invert_prof,phy27 | 55 | 0,01 | 1 | 0,81 | 0,93 | 0,25 | 0,0618 | 18,18 | 21,82 | 20 | 12,73 | 27,27 |
| my_IRR_IGF1R_IR_Invert_prof,phy28 | 55 | 0 | 1 | 0,83 | 0,93 | 0,25 | 0,0606 | 16,36 | 16,36 | 21,82 | 18,18 | 27,27 |
| my_IRR_IGF1R_IR_Invert_prof,phy29 | 55 | 0 | 1 | 0,84 | 0,93 | 0,24 | 0,056 | 12,73 | 21,82 | 20 | 18,18 | 27,27 |
| my_IRR_IGF1R_IR_Invert_prof,phy2 | 55 | 0,01 | 1 | 0,79 | 0,93 | 0,29 | 0,0852 | 21,82 | 12,73 | 21,82 | 18,18 | 25,45 |
| my_IRR_IGF1R_IR_Invert_prof,phy30 | 55 | 0,01 | 1 | 0,8 | 0,94 | 0,28 | 0,0756 | 23,64 | 12,73 | 20 | 18,18 | 25,45 |
| my_IRR_IGF1R_IR_Invert_prof,phy31 | 55 | 0,01 | 1 | 0,82 | 0,93 | 0,25 | 0,0632 | 18,18 | 14,55 | 23,64 | 16,36 | 27,27 |
| my_IRR_IGF1R_IR_Invert_prof,phy32 | 55 | 0,02 | 1 | 0,81 | 0,94 | 0,27 | 0,0723 | 23,64 | 10,91 | 20 | 18,18 | 27,27 |
| my_IRR_IGF1R_IR_Invert_prof,phy33 | 55 | 0,01 | 1 | 0,8 | 0,94 | 0,31 | 0,0936 | 20 | 14,55 | 18,18 | 18,18 | 29,09 |
| my_IRR_IGF1R_IR_Invert_prof,phy34 | 55 | 0,01 | 1 | 0,79 | 0,93 | 0,29 | 0,0843 | 23,64 | 14,55 | 21,82 | 12,73 | 27,27 |
| my_IRR_IGF1R_IR_Invert_prof,phy35 | 55 | 0,12 | 1 | 0,83 | 0,94 | 0,23 | 0,0521 | 21,82 | 10,91 | 20 | 18,18 | 29,09 |
| my_IRR_IGF1R_IR_Invert_prof,phy36 | 55 | 0 | 1 | 0,8 | 0,93 | 0,28 | 0,0778 | 21,82 | 12,73 | 23,64 | 12,73 | 29,09 |
| my_IRR_IGF1R_IR_Invert_prof,phy37 | 55 | 0,01 | 1 | 0,79 | 0,94 | 0,29 | 0,0859 | 21,82 | 16,36 | 21,82 | 14,55 | 25,45 |
| my_IRR_IGF1R_IR_Invert_prof,phy38 | 55 | 0,02 | 1 | 0,82 | 0,93 | 0,25 | 0,0612 | 20 | 12,73 | 25,45 | 14,55 | 27,27 |
| my_IRR_IGF1R_IR_Invert_prof,phy39 | 55 | 0,03 | 1 | 0,78 | 0,93 | 0,29 | 0,0851 | 25,45 | 9,09 | 23,64 | 14,55 | 27,27 |
| my_IRR_IGF1R_IR_Invert_prof,phy3 | 55 | 0,01 | 1 | 0,82 | 0,93 | 0,25 | 0,0645 | 20 | 10,91 | 25,45 | 16,36 | 27,27 |
| my_IRR_IGF1R_IR_Invert_prof,phy40 | 55 | 0,01 | 1 | 0,82 | 0,94 | 0,25 | 0,0613 | 16,36 | 23,64 | 16,36 | 14,55 | 29,09 |
| my_IRR_IGF1R_IR_Invert_prof,phy4 | 55 | 0,17 | 1 | 0,82 | 0,94 | 0,25 | 0,0602 | 20 | 10,91 | 21,82 | 20 | 27,27 |
| my_IRR_IGF1R_IR_Invert_prof,phy5 | 55 | 0,04 | 1 | 0,83 | 0,94 | 0,23 | 0,0507 | 18,18 | 20 | 18,18 | 16,36 | 27,27 |
| my_IRR_IGF1R_IR_Invert_prof,phy6 | 55 | 0 | 1 | 0,81 | 0,94 | 0,28 | 0,0757 | 21,82 | 10,91 | 25,45 | 16,36 | 25,45 |
| my_IRR_IGF1R_IR_Invert_prof,phy7 | 55 | 0,02 | 1 | 0,78 | 0,93 | 0,3 | 0,0911 | 25,45 | 9,09 | 21,82 | 18,18 | 25,45 |
| my_IRR_IGF1R_IR_Invert_prof,phy8 | 55 | 0,12 | 1 | 0,85 | 0,93 | 0,22 | 0,0503 | 14,55 | 14,55 | 25,45 | 16,36 | 29,09 |
| my_IRR_IGF1R_IR_Invert_prof,phy9 | 55 | 0,01 | 1 | 0,8 | 0,94 | 0,28 | 0,0807 | 23,64 | 12,73 | 20 | 14,55 | 29,09 |
| my_IRR_IGF1R_IR_Invert_prof,phy_NJ | 55 | 0 | 1 | 0,79 | 0,93 | 0,3 | 0,0925 | 21,82 | 10,91 | 21,82 | 16,36 | 29,09 |
| | mean | 0,04804 | 1 | 0,81634 | 0,93414 | 0,256585 | 0,06637804 | 19,955853 | 14,36975 | 21,463170 | 16,496585 | 27,713902 |
| | median | 0,01 | 1 | 0,82 | 0,93 | 0,25 | 0,0638 | 20 | 14,55 | 21,82 | 16,36 | 27,27 |

The data indicate a good overall tree resolution, with a mean median support value of 0.93, calculated over the 41 trees. Forty four percent of the cumulative 2255 bipartitions in the sample had a significant support value ($P \geq 0.95$), and 65% of them had a ($P \geq 0.85$). Notice that means and median values are very similar. The range of model ln*L* values was: 30836.65814 - 30870.89200, only 34 units of difference. The highest-scoring tree highlighted by shading.

## C) Shimodaira-Hasegawa comparison of 41 user-supplied PhyML trees.

Fourty of them used random sequential addition seed trees and the remaining one a (default) BioNJ starter phylogeny.
All were run with PhyML 2.4.5 under the best-approximating JTT+G model.
Analysis performed with Puzzle 5.2 [3] using the randomly chosen tree #6 for model parameter estimation.

| Tree | log L | difference | S.E. | p-1sKH | p-SH | c-ELW | 2sKH | |
|---|---|---|---|---|---|---|---|---|
| 1 | -30920.22 | 19.13 | 28.9019 | 0.2540 + | 0.8270 + | 0.0032 - | + | |
| 2 | -30918.11 | 17.02 | 32.7880 | 0.2980 + | 0.8130 + | 0.0195 + | + | |
| 3 | -30921.04 | 19.95 | 28.6171 | 0.2240 + | 0.7840 + | 0.0049 - | + | |
| 4 | -30909.99 | 8.90 | 18.1504 | 0.3060 + | 0.9380 + | 0.0179 + | + | |
| 5 | -30916.65 | 15.56 | 26.1724 | 0.2780 + | 0.8540 + | 0.0187 + | + | |
| 6 | -30901.09 | 0.00 | <---- best | 1.0000 + | 1.0000 + | 0.1544 + | best | |
| 7 | -30933.54 | 32.46 | 29.8335 | 0.1520 + | 0.5110 + | 0.0000 - | + | |
| 8 | -30918.16 | 17.07 | 31.9574 | 0.2930 + | 0.8390 + | 0.0090 + | + | |
| 9 | -30921.24 | 20.15 | 21.7312 | 0.1810 + | 0.7080 + | 0.0326 + | + | |
| 10 | -30911.82 | 10.73 | 23.0560 | 0.3120 + | 0.8600 + | 0.0621 + | + | |
| 11 | -30926.76 | 25.67 | 32.6045 | 0.2020 + | 0.6290 + | 0.0089 + | + | |
| 12 | -30928.11 | 27.02 | 31.5468 | 0.2130 + | 0.6390 + | 0.0042 - | + | |
| 13 | -30925.87 | 24.78 | 30.1233 | 0.2080 + | 0.6750 + | 0.0076 + | + | |
| 14 | -30918.64 | 17.55 | 28.5998 | 0.2500 + | 0.8370 + | 0.0160 + | + | |
| 15 | -30915.19 | 14.11 | 27.9301 | 0.3000 + | 0.8820 + | 0.0120 + | + | **rdm tree#15** |
| 16 | -30928.55 | 27.46 | 29.0529 | 0.1750 + | 0.5880 + | 0.0183 + | + | |
| 17 | -30910.53 | 9.45 | 19.1169 | 0.3160 + | 0.9290 + | 0.0271 + | + | |
| 18 | -30923.15 | 22.06 | 29.9950 | 0.2360 + | 0.7320 + | 0.0083 + | + | |
| 19 | -30922.78 | 21.69 | 28.0707 | 0.2340 + | 0.7210 + | 0.0204 + | + | |
| 20 | -30927.55 | 26.46 | 31.0046 | 0.1940 + | 0.6410 + | 0.0084 + | + | |
| 21 | -30905.19 | 4.10 | 22.8222 | 0.4290 + | 0.9470 + | 0.1050 + | + | |
| 22 | -30913.79 | 12.70 | 22.0795 | 0.2720 + | 0.8750 + | 0.0129 + | + | |
| 23 | -30910.28 | 9.19 | 19.0054 | 0.2990 + | 0.9390 + | 0.0187 + | + | |
| 24 | -30922.79 | 21.70 | 29.3545 | 0.2400 + | 0.7660 + | 0.0036 - | + | |
| 25 | -30922.89 | 21.81 | 31.2019 | 0.2660 + | 0.7270 + | 0.0087 + | + | |
| 26 | -30930.51 | 29.42 | 32.5653 | 0.1780 + | 0.5560 + | 0.0054 - | + | |
| 27 | -30916.59 | 15.50 | 28.0001 | 0.2800 + | 0.8600 + | 0.0069 - | + | |
| 28 | -30912.32 | 11.23 | 16.7926 | 0.2360 + | 0.9110 + | 0.0065 - | + | |
| 29 | -30925.54 | 24.45 | 22.9266 | 0.1330 + | 0.6980 + | 0.0026 - | + | |
| 30 | -30925.80 | 24.71 | 23.5154 | 0.1600 + | 0.7310 + | 0.0012 - | + | |
| 31 | -30918.39 | 17.30 | 29.2052 | 0.2850 + | 0.8420 + | 0.0113 + | + | |
| 32 | -30917.78 | 16.69 | 27.6657 | 0.2900 + | 0.8420 + | 0.0116 + | + | |
| 33 | -30913.84 | 12.75 | 27.6496 | 0.3250 + | 0.9200 + | 0.0167 + | + | |
| 34 | -30907.55 | 6.46 | 21.4700 | 0.3640 + | 0.9500 + | 0.0450 + | + | |
| 35 | -30918.02 | 16.93 | 30.6274 | 0.2670 + | 0.8320 + | 0.0123 + | + | |
| 36 | -30911.59 | 10.50 | 23.7767 | 0.3380 + | 0.8860 + | 0.0444 + | + | |
| 37 | -30910.64 | 9.55 | 28.3319 | 0.3550 + | 0.8650 + | 0.0840 + | + | |
| 38 | -30925.24 | 24.15 | 29.6290 | 0.2140 + | 0.6880 + | 0.0047 - | + | |
| 39 | -30911.88 | 10.79 | 26.1383 | 0.3220 + | 0.8780 + | 0.0620 + | + | |
| 40 | -30924.83 | 23.74 | 35.7654 | 0.2460 + | 0.6610 + | 0.0144 + | + | |
| 41 | -30909.60 | 8.51 | 20.2645 | 0.3250 + | 0.8920 + | 0.0685 + | + | **BioNJ tree** |

The columns show the results and p-values of the following tests:
**1sKH** - one sided KH test based on pairwise SH tests
**SH** - Shimodaira-Hasegawa test (1999) [2]
**ELW** - Expected Likelihood Weight (Strimmer-Rambaut 2002)[4]
**2sKH** - two sided Kishino-Hasegawa test (1989)[1].

Plus signs denote the confidence sets. Minus signs denote significant exclusion. All tests used 5% significance level. 1sKH, SH, and ELW performed 1000 resamplings using the RELL method.
1sKH and 2sKH are correct to the 2nd position after the the decimal point of the log-likelihoods.

As shown by the table above, all competing trees were congruent when evaluated by the very conservative Shimodaira-Hasegawa test. However, the more stringent expected likelihood weights test uncovered 11 significantly incongruent topologies. The ln*L* scores of the best tree (sarted from random seed tree #15) and that one found starting from the defuaul BioNJ tree are not significantly different.

## D) PhyML tree search statistics.

**D1)** Sorted log likelihood scores for the ML trees obtained for each individual tree search initiated from a random tree (phy*_phyml) vs. that obtained for the standard search initiated from a BioNJ tree.

```
-30836.65814 = my_IRR_IGF1R_IR_Invert_prof.phy15_phyml_stat.txt
-30840.27766 = my_IRR_IGF1R_IR_Invert_prof.phy29_phyml_stat.txt
-30841.18697 = my_IRR_IGF1R_IR_Invert_prof.phy40_phyml_stat.txt
-30844.51886 = my_IRR_IGF1R_IR_Invert_prof.phy_NJ_phyml_stat.txt
-30845.52360 = my_IRR_IGF1R_IR_Invert_prof.phy6_phyml_stat.txt
-30845.70280 = my_IRR_IGF1R_IR_Invert_prof.phy13_phyml_stat.txt
-30845.76546 = my_IRR_IGF1R_IR_Invert_prof.phy19_phyml_stat.txt
-30845.88096 = my_IRR_IGF1R_IR_Invert_prof.phy25_phyml_stat.txt
-30845.91526 = my_IRR_IGF1R_IR_Invert_prof.phy30_phyml_stat.txt
-30846.11650 = my_IRR_IGF1R_IR_Invert_prof.phy5_phyml_stat.txt
-30848.04128 = my_IRR_IGF1R_IR_Invert_prof.phy35_phyml_stat.txt
-30848.57785 = my_IRR_IGF1R_IR_Invert_prof.phy2_phyml_stat.txt
-30849.78780 = my_IRR_IGF1R_IR_Invert_prof.phy8_phyml_stat.txt
-30851.61455 = my_IRR_IGF1R_IR_Invert_prof.phy3_phyml_stat.txt
-30852.35625 = my_IRR_IGF1R_IR_Invert_prof.phy14_phyml_stat.txt
-30854.20135 = my_IRR_IGF1R_IR_Invert_prof.phy23_phyml_stat.txt
-30854.57105 = my_IRR_IGF1R_IR_Invert_prof.phy17_phyml_stat.txt
-30854.93661 = my_IRR_IGF1R_IR_Invert_prof.phy34_phyml_stat.txt
-30855.36022 = my_IRR_IGF1R_IR_Invert_prof.phy38_phyml_stat.txt
-30855.83467 = my_IRR_IGF1R_IR_Invert_prof.phy22_phyml_stat.txt
-30856.37565 = my_IRR_IGF1R_IR_Invert_prof.phy39_phyml_stat.txt
-30857.06373 = my_IRR_IGF1R_IR_Invert_prof.phy18_phyml_stat.txt
-30857.07095 = my_IRR_IGF1R_IR_Invert_prof.phy11_phyml_stat.txt
-30857.25147 = my_IRR_IGF1R_IR_Invert_prof.phy10_phyml_stat.txt
-30857.59641 = my_IRR_IGF1R_IR_Invert_prof.phy4_phyml_stat.txt
-30860.00784 = my_IRR_IGF1R_IR_Invert_prof.phy27_phyml_stat.txt
-30860.29513 = my_IRR_IGF1R_IR_Invert_prof.phy31_phyml_stat.txt
-30860.44945 = my_IRR_IGF1R_IR_Invert_prof.phy12_phyml_stat.txt
-30860.49945 = my_IRR_IGF1R_IR_Invert_prof.phy26_phyml_stat.txt
-30861.82375 = my_IRR_IGF1R_IR_Invert_prof.phy21_phyml_stat.txt
-30862.07270 = my_IRR_IGF1R_IR_Invert_prof.phy7_phyml_stat.txt
-30862.30288 = my_IRR_IGF1R_IR_Invert_prof.phy9_phyml_stat.txt
-30862.53409 = my_IRR_IGF1R_IR_Invert_prof.phy37_phyml_stat.txt
-30862.77819 = my_IRR_IGF1R_IR_Invert_prof.phy32_phyml_stat.txt
-30863.40874 = my_IRR_IGF1R_IR_Invert_prof.phy36_phyml_stat.txt
-30864.61757 = my_IRR_IGF1R_IR_Invert_prof.phy24_phyml_stat.txt
-30865.27667 = my_IRR_IGF1R_IR_Invert_prof.phy1_phyml_stat.txt
-30866.44167 = my_IRR_IGF1R_IR_Invert_prof.phy33_phyml_stat.txt
-30866.98699 = my_IRR_IGF1R_IR_Invert_prof.phy28_phyml_stat.txt
-30867.14938 = my_IRR_IGF1R_IR_Invert_prof.phy20_phyml_stat.txt
-30870.89200 = my_IRR_IGF1R_IR_Invert_prof.phy16_phyml_stat.txt
```

**D2)** Frequency of the ln*L* scores.

```
-30836.65814 = 1
-30840.27766 = 1
-30841.18697 = 1
-30844.51886 = 1
-30845.52360 = 1
-30845.70280 = 1
-30845.76546 = 1
-30845.88096 = 1
-30845.91526 = 1
-30846.11650 = 1
-30848.04128 = 1
-30848.57785 = 1
-30849.78780 = 1
-30851.61455 = 1
-30852.35625 = 1
-30854.20135 = 1
-30854.57105 = 1
-30854.93661 = 1
-30855.36022 = 1
-30855.83467 = 1
-30856.37565 = 1
-30857.06373 = 1
-30857.07095 = 1
-30857.25147 = 1
-30857.59641 = 1
-30860.00784 = 1
-30860.29513 = 1
-30860.44945 = 1
-30860.49945 = 1
-30861.82375 = 1
-30862.07270 = 1
-30862.30288 = 1
-30862.53409 = 1
-30862.77819 = 1
-30863.40874 = 1
-30864.61757 = 1
-30865.27667 = 1
-30866.44167 = 1
-30866.98699 = 1
-30867.14938 = 1
-30870.89200 = 1
```

These data show that three trees were found that had a better score than that found by the default PhyML search (starting from a BioNJ seed tree), although not significantly better, as shown by the SH and c-ELW tests above. The fact that as many tree islands were found as searches were started indicates it gets stuck in different peaks that are in a narrow height range of ln*L* scores: -30836.65814 (best) to -30870.89200 (worst).

**References not cited in the paper:**


[1] Kishino H, Hasegawa M (1989) Evaluation of the maximum likelihood estimate of the evolutionary tree topologies from DNA sequence data, and the branching order in hominoidea. J Mol Evol 29(2): 170-179.

[2] Shimodaira H, Hasegawa M (1999) Multiple comparisons of log-likelihoods with applications to phylogenetic inference. Mol Biol Evol 16: 1114-1116.

[3] Schmidt HA, Strimmer K, Vingron M, von Haeseler A (2002) TREE-PUZZLE: maximum likelihood phylogenetic analysis using quartets and parallel computing. Bioinformatics 18(3): 502-504.

[4] Strimmer K, Rambaut A (2002) Inferring confidence sets of possibly misspecified gene trees. Proc Biol Sci 269(1487): 137-142.
