# Supplementary material for: A Comparative Structural Bioinformatics Analysis of the Insulin Receptor Family Ectodomain Based on Phylogenetic Information
Source: PLoS One. 2008 Nov 7;3(11):e3667. doi: 10.1371/journal.pone.0003667 (PMC2577065; doi:10.1371/journal.pone.0003667)
Supplement: Supplementary File S2 — Supplemental Information for the Bayesian Analyses (0.65 MB PDF) [file pone.0003667.s002.pdf]

Bayesian Analysis of the the 55 vertebrate ectodomain sequences listed in Table 1, plus three IR family invertebrate homologous ectodomain sequences.

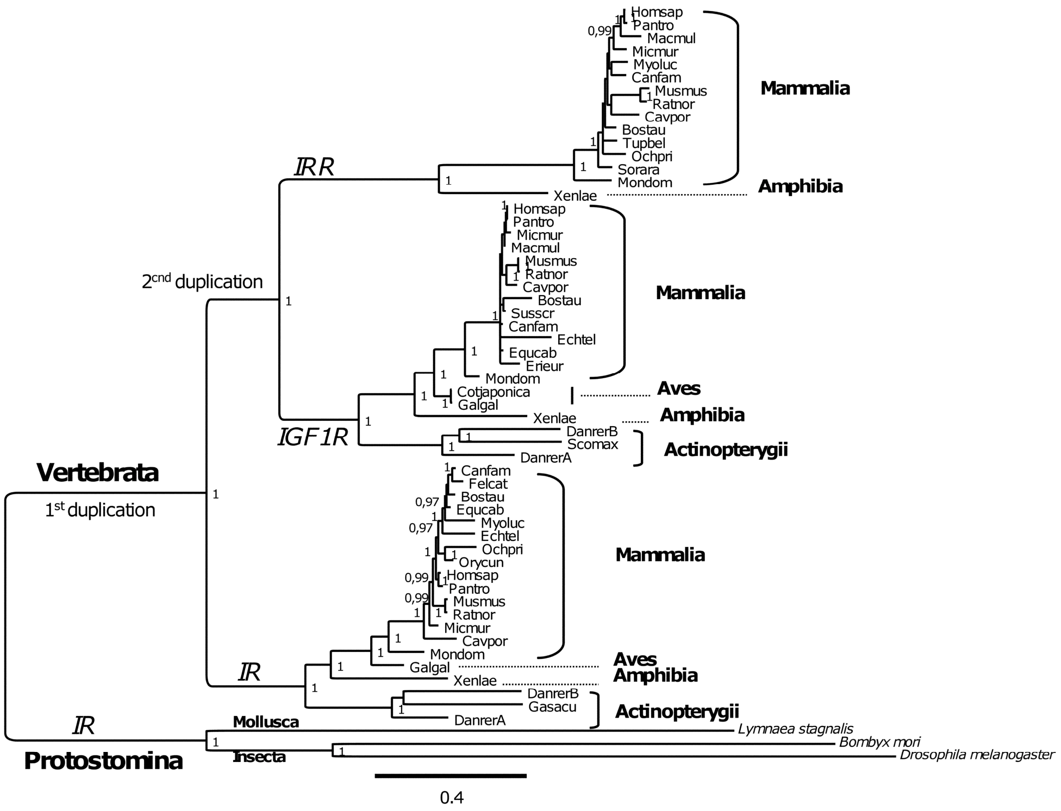

Figure S1. Bayesian phylogeny of the IR family.

Bayesian phylogeny of the IR family ectodomain inferred from amino acid sequences. Two independent MC3 runs, each replicated twice, yielded identical topologies, indicating excellent convergence. The numbers at the bipartitons correspond to their posterior probabilities (only shown if ≥ 0.95), estimated from their proportion of occurrences among 8002 source trees (4001 x 2, after discarding the first 1000 trees of each run as burnin) used to calculate the majority rule consensus tree shown. The scale indicates the number of expected substitutions per site under the best fitting JTT+G model (shape parameter α = 0.96), which had a posterior probability of 1. Compare this figure with Fig. 1 in the paper, which shows the corresponding ML phylogeny.

Statistical analysis of the four Bayesian MC^3 runs.

Generation plots of ln*L* values for the four Bayesian MC^3 runs. Notice the good mixing and convergence of the individual runs

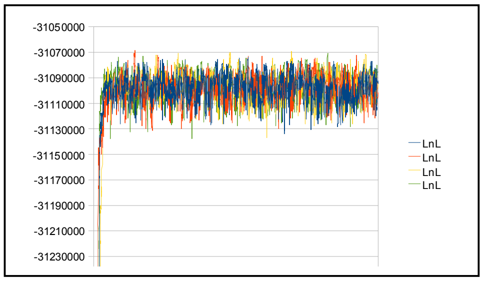

Generation plots of tree length values for the four Bayesian MC^3 runs. Notice the good mixing and convergence of the individual runs

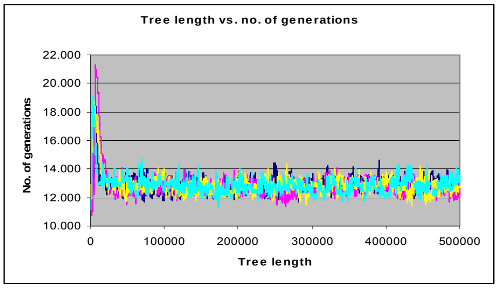

Estimated marginal likelihoods for replicate 1 and 2 of *Run1*:

Rep	Arithmetic mean	Harmonic mean
1	-31078.29	-31129.18
2	-31078.93	-31123.45
TOTAL	-31078.56	**-31128.49**

Estimated marginal likelihoods for replicate 1 and 2 of *Run2*:

Rep	Arithmetic mean	Harmonic mean
1	-31076.18	-31128.40
2	-31080.54	-31125.36
TOTAL	-31076.86	**-31127.75**

Model parameter summaries over the replicate 1 and 2 of *Run1*:

Summaries are based on a total of 8002 samples from 2 replicated runs. Each run produced 5001 samples of which 4001 samples were included.

Parameter	Mean	Variance	95% Cred. Interval Lower	Upper	Median	PSRF *
TL	12.766011	0.196148	11.965000	13.689000	12.751000	1.017
alpha	0.960459	0.002124	0.873029	1.053529	0.958775	1.000

* Convergence diagnostic (PSRF = Potential scale reduction factor [Gelman and Rubin, 1992], uncorrected) should approach 1 as runs converge. The values may be unreliable if you have a small number of samples. PSRF should only be used as a rough guide to convergence since all the assumptions that allow one to interpret it as a scale reduction factor are not met in the phylogenetic context.

Model parameter summaries over the replicate 1 and 2 of *Run2*:

Summaries are based on a total of 8002 samples from 2 replicated runs. Each run produced 5001 samples of which 4001 samples were included.

Parameter	Mean	Variance	95% Cred. Interval Lower	Upper	Median	PSRF *
TL	12.726446	0.183333	11.876000	13.535000	12.739000	1.008
alpha	0.962038	0.002120	0.875532	1.054901	0.961335	1.001

* Convergence diagnostic (PSRF = Potential scale reduction factor [Gelman and Rubin, 1992], uncorrected) should approach 1 as runs converge. The values may be unreliable if you have a small number of samples. PSRF should only be used as a rough guide to convergence since all the assumptions that allow one to interpret it as a scale reduction factor are not met in the phylogenetic context.

Model selection using reversible jump MC^3 replicate 1 and 2 of *Run1*:

Amino acid model probabilities:

Model	Posterior Probability	Standard Deviation
Poisson	0.000	0.000000
**Jones**	**1.000**	**0.000000**
Dayhoff	0.000	0.000000
Mtrev	0.000	0.000000
Mtmam	0.000	0.000000
Wag	0.000	0.000000
Rtrev	0.000	0.000000
Cprev	0.000	0.000000
Vt	0.000	0.000000
Blosum	0.000	0.000000

Model selection using reversible jump MC^3 replicate 1 and 2 of *Run2*:

Amino acid model probabilities:

Model	Posterior Probability	Standard Deviation
Poisson	0.000	0.000000
**Jones**	**1.000**	**0.000000**
Dayhoff	0.000	0.000000
Mtrev	0.000	0.000000
Mtmam	0.000	0.000000
Wag	0.000	0.000000
Rtrev	0.000	0.000000
Cprev	0.000	0.000000
Vt	0.000	0.000000
Blosum	0.000	0.000000
